# Detection of Simulated Space Weathering on Electron Irradiated Water-Ice Coated Silicon Using AFM-IR, SEM, and S/TEM Techniques

**DOI:** 10.1021/acsearthspacechem.6c00135

**Published:** 2026-06-27

**Authors:** Caroline E. Caplan, Hope A. Ishii, Jeffrey J. Gillis, Kevin D. McKeegan, Ming-Chang Liu, Gerardo Dominguez

**Affiliations:** † Physics Department, 14673 California State University, San Marcos, California 92096, United States; ‡ Hawai‘i Institute of Geophysics and Planetology, 3949 University of Hawai‘i Ma̅noa, Honolulu, Hawaii 96822, United States; § Department of Physics, 7548 Washington University in St. Louis, St. Louis, Missouri 63130, United States; ∥ Department of Earth, Planetary, and Space Sciences, 8783 University of California, Los Angeles, California 90095, United States

**Keywords:** electron irradiation, oxidation, space weathering, water-ice, AFM-IR, cosmic rays, IR spectroscopy

## Abstract

Space weathering causes physical and spectral changes on the surfaces of airless bodies. However, our understanding of how space weathering operates in the presence of volatile ices is in its early stages. Electron irradiation of ice-coated surfaces is expected in astrophysical environments including the early solar system, volatile ice-rich permanently shadowed regions of the Moon and Mercury, and other airless bodies like asteroids. A recent study suggests that anomalous oxygen isotope exchange occurs between water-ice and underlying surfaces when exposed to electron irradiation at extremely low temperatures (10 K). To delve deeper into the physical processes underlying isotopic exchange, we employ nanoscale atomic force microscopy-based infrared (AFM-IR) spectroscopy to identify Si–O bond formation resulting from the electron irradiation of H_2_O ice coated silicon targets. Experimental variables include electron energy, amount and timing of water-ice deposition, and surface area exposed to the electron beam. AFM-IR point spectra, surface topography and IR absorption mapping reveal that the degree of surface oxidation is dependent upon experimental conditions. Scanning electron microscopy and (scanning) transmission electron microscope imaging confirm the formation of thicker SiO_*x*_ in regions of enhanced interaction between electron irradiation, water-ice, and the silicon substrate. In summary, we find that electron irradiation with energies as low as 1 keV/electron can break the chemical bonds of refractory solids like Si under these simulated cold astrophysical conditions. These results suggest that cosmic rays may play a more significant role than previously thought in the chemical evolution of dust grains in cold astrophysical and protoplanetary environments.

## Graphical abstract

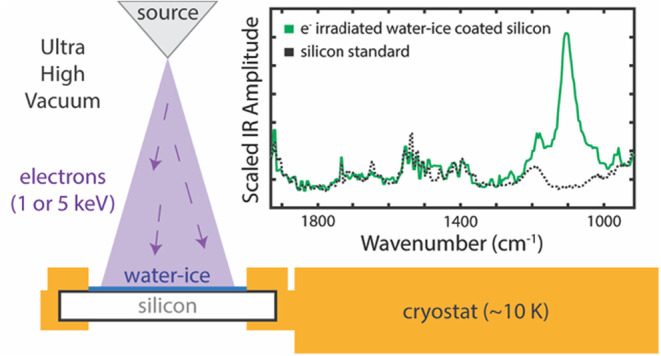

## Introduction

Space weathering processes cause physical and chemical changes on the surfaces of airless bodies. These changes result from various interactions, such as impacts of micrometeoroids, as well as exposure to ionized particles that constitute the solar wind and galactic cosmic rays (CRs).^1–3^ The repeated interactions of meteoroids of all sizes comminute and garden planetary bodies surfaces. Solar wind and galactic CR irradiation affect surfaces differently, leading to the internal rearrangement or release of atoms near the surface of a grain via sputtering, depending on their energy and corresponding penetration depths.^3,4^ These irradiation sources are dominated by the fluxes of protons, electrons, and some heavier nuclei, each with different characteristic energies that range from ∼1 eV to >10 GeV and penetration depths from micrometer to meter.^1,5–8^

Chemical changes experienced by solid surfaces on airless bodies result from multiple processes that occur when high-energy ionized particles (solar and galactic CR) penetrate and decelerate in solids. First, these high-energy particles ionize atoms and molecules of the material by stripping electrons from the atoms of the solid via electrostatic interactions between the incoming nuclei and the electrons found in the material.^9^ This ionization also transfers kinetic energy to the electrons, which leads to heating of the material. Second, atom displacement drives defects in crystal structures. These dislocations occur either through direct collisions or collisions with secondary electrons, atoms, or ions. Finally, at energies ≥MeV, nuclear reactions occur between high-energy particles and atomic nuclei within the material. These reactions may produce new isotopes and daughter products, along with secondary particles, such as neutrons, pions, and γ rays.^1,10,11^

The potential role of space weathering by energetic particles in altering the oxygen isotopic composition of solids in the solar system remains an area of active investigation. Recent experimental work has shown that space weathering by energetic electrons of water-ice coated surfaces results in shifts in the isotopic composition of metal oxide surfaces (SiO_4_, Al_2_O_3_).^12^ These shifts, however, are not uniformly distributed on the experimental surfaces. One of the goals of this work is to better understand these surface isotopic heterogeneities by studying how similar experiments on Si might lead to heterogeneous patterns of surface oxidation. Additionally, we aim to better understand how electron irradiation in the presence of volatile water ice can affect refractory solids.

Here we report on a set of experiments using Si substrates, similar to those in previous work on SiO_2_ substrates by Dominguez et al. (2019 and under review)^12^ that vary electron energy, volume of water-ice deposition, surface area exposed to the electron beam, and timing of water-ice deposition. Using pure silicon wafers removes any ambiguity in detecting chemical changes on a solid surface induced by electron irradiation and the presence of water ice. This ambiguity is removed because pure silicon lacks IR absorption features in the range where Si–O bond peaks appear (1000–1200 cm^–1^), dominantly around 1100 cm^–1^.^13,14^

Using Atomic Force Microscopy based-infrared spectroscopy (AFM-IR), we detected heterogeneous surface oxidation that depended on the experimental conditions. Additionally, we observed changes in the surface topography of the Si surfaces using Scanning Electron Microscopy (SEM), (scanning) transmission electron microscope (S/TEM), and AFM-IR imaging.

## Methods

### Samples and Preparation

Each silicon disk was cut from a single silicon wafer (Ted Pella no. 16004) using a diamond-tipped plug cutter drill bit and distilled water as a coolant and to contain silicon dust in the drilling process, producing 12.4 mm diameter disks (3.2 mm thickness). These were cleaned with methanol afterward using Kimwipe tissues (Kimberly-Clark). The top of each sample was inscribed with a V-shape using a diamond tip scribe to ensure surface locations could be tracked throughout analyses (see Figure S1 in Supporting Information).

Some of these silicon wafers were set aside as in-house baseline controls and were measured using AFM-IR to aid our interpretation of the experimental data. Two chips were used as controls and examined throughout different AFM-IR sessions to determine if oxidation occurred via atmospheric exposure. One chip–referred to as *Silicon Control*–was kept in a vacuum desiccator along with the experiment samples, and the other chip–*Atmosphere Control*–was stored in a sample drawer exposed to air. Additionally, a Si/SiO_2_ grating sample (Ted Pella no. 629–10AFM) was used to compare with experimental samples and the Si in-house controls.

### Experimental Setup

Electron irradiation of water-ice coated silicon disk substrates was achieved using the Isotopic Characterization Experiment (ICE) at California State University San Marcos (CSUSM) (Figure 1). For each experiment, a silicon disk was mounted inside the circular well of the gold-plated sample holder (lined with indium wire to help secure the sample) that was attached to a cryostat (ColdEdge, model number CH-204SFF). The disk was secured with a gold-plated ring cover and ultrahigh vacuum (UHV) rated screws. UHV of ∼1 × 10^–8^ Torr was achieved using an oil free scroll pump and a magnetically levitated turbo pump (Shimadzu, model number TMP-303LMC). To create ice, the cryostat was set to 10 K using a temperature control unit (Lakeshore 336 Temperature Controller) which resulted in injected water vapor freezing out onto the cold surface. We note that the silicon diode thermistor used for temperature monitoring and control is mounted at the edge of the target, and it is possible that the center of a sample may have warmed due to the energy deposition from the electron beam during experiments.

**Figure 1:**
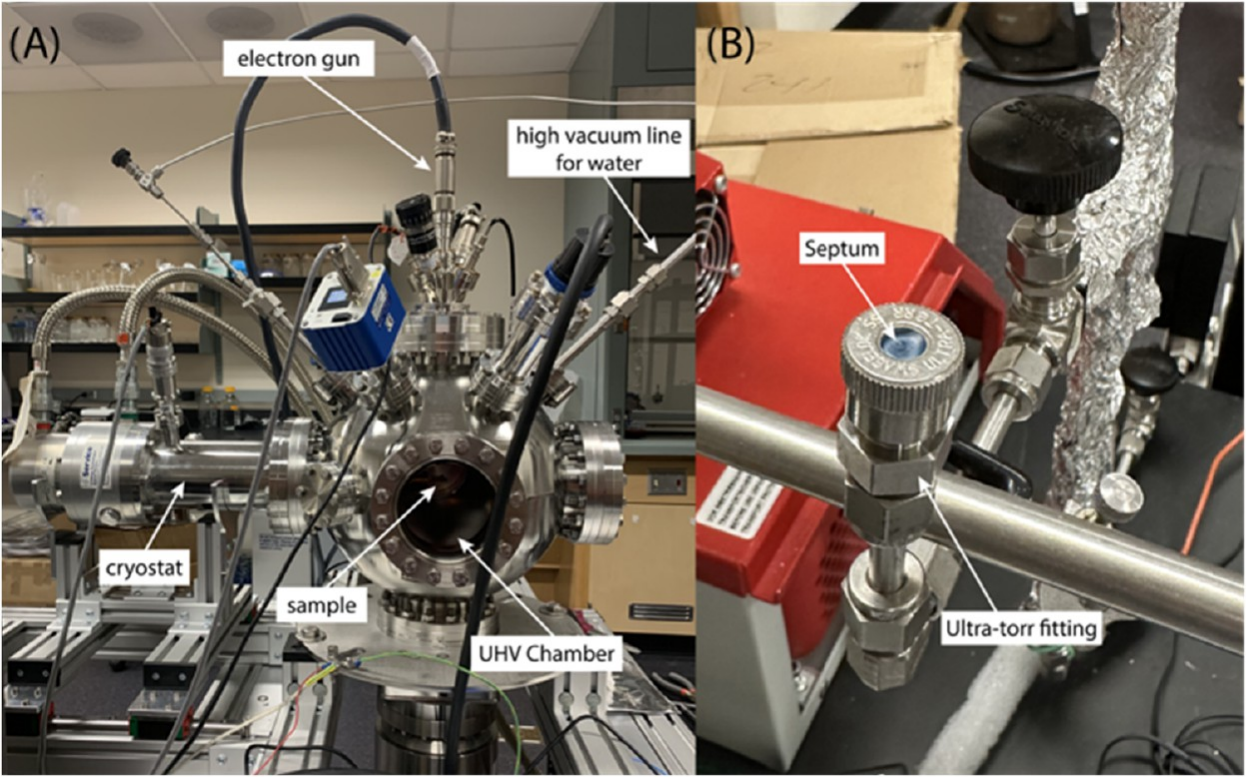
Setup for the Isotopic Characterization Experiment (ICE) at CSUSM. (A) The UHV chamber houses the sample disk which is mounted into the arm of the cryostat and pumped down to ∼1 × 10^–8^ Torr. The electron gun is aimed at the sample from above. (B) The water standard is injected into a high vacuum line via an ultratorr fitting with a septum. This vacuum line is pointed directly at the sample allowing water to be deposited onto the cold sample surface.

Greenland Ice Sheet Precipitation (GISP) from the National Institutes of Standards and Technology (NIST) was the chosen water standard for these experiments. The water standard was injected into a high-vacuum line (HVL) first by using a stainless-steel syringe via a port with a custom modified septum (Restek IceBlue 9.5 mm no. 27159) ultratorr fitting (Swagelok no. SS-4-UT-6–400) (Figure 1b). The water vapor was deposited onto the sample via a valved stainless-steel pipe that is pointed directly at the sample surface within the chamber. Due to the system setup, water was added in increments of either 10 or 5 μL approximately 25 min apart, and the pressure in the HVL was used to confirm successful injection of specified water aliquots. The water standard was deposited on the surface of a sample in the same manner for each experiment, but in varying quantities and at different timings relative to irradiation. In order to protect the turbo pump, the UHV chamber gate valve was closed, and the turbo pump was turned off before experiments began, both before irradiation or water deposition onto the silicon substrate. The scroll pump continued to run during experiments to help minimize air leakage across the gate valve.

We completed a range of experiments for this study (Table 1): (1) *pre-*irradiation experiments in which the water standard was deposited onto a silicon substrate before irradiation began (named *5 keV pre-10, -25, -50 μL, and 1 keV pre-25, -10 μL 1/3 surface area (SA))*, (2) *post-*irradiation experiments in which the water standard was deposited after irradiation ended to determine if water needs to be simultaneously present for oxidation to occur (named *5 keV* and *1 keV post-25 μL*), and (3) control experiments for irradiation and water deposition in which one was conducted without water-ice (named *Irradiation Only*) and one with 25 μL of water standard deposition without any irradiation (named *Water-Ice Only*).

**Table 1:** Conditions for Electron Irradiation Experiments and Controls

Sample	Water (μL)	Duration (min)	Average Flux (×10^14^ electrons cm^–2^ s^–1^)	Energy per Electron (eV)	Energy Deposited (×10^22^ eV)	Beam Radius (mm)	Energy per Unit Area (×10^20^ eV mm^–2^)
Controls^a^	-	-	-	-	-	-	-
Water-Ice Only	25	450	-	-	-	-	-
Irradiation Only	-	460	6.87	5000	4.22	3.77	9.48
5 keV pre-10 μL	10	430	7.36	5000	4.23	3.77	9.50
5 keV pre-25 μL	25	460	6.98	5000	4.29	3.77	9.64
5 keV pre-50 μL	50	430	7.47	5000	4.29	3.77	9.64
5 keV post-25 μL	25	550	5.79	5000	4.25	3.77	9.56
1 keV pre-25 μL	25	570	5.47	1000	0.84	3.79	1.87
1 keV post-25 μL	25	480	5.84	1000	0.85	4.02	1.68
1 keV pre-10 μL^b^	10	420	23.5	1000	0.85	2.14	5.92

a Unexposed controls include the Silicon Control, Atmosphere Control, and the Si/SiO_2_ Grating.

b One third (1/3) the electron beam surface area (SA) compared to other experiments.

For the *pre-*irradiation experiments in which water was deposited before irradiation began, the irradiation component of the experiments was conducted when the UHV chamber stabilized to a pressure of ∼2 × 10^–8^ Torr after water standard deposition. After irradiation, the electron gun was turned off, and after approximately 30 min, the cryostat was turned off. The waiting period allowed the electron source filament to cool down before water-ice volatilized in the chamber as the sample and surrounding cold surfaces returned to room temperature.

For the *post-*irradiation experiments in which water was deposited after irradiation ended, the electron gun was turned off for 30 min before introducing water vapor into the chamber (same method as above) to help prolong the lifespan of the filament. The cryostat was turned off approximately 25 min after the final increment of water was deposited to ensure maximal freeze out of water vapor.

### Electron Irradiation

A Kimball Physics electron gun (EGPS-2017C) was used to irradiate the silicon samples with energetic electrons. The beam was set to an energy of 1 or 5 keV/electron and a current of ∼50 μA for ∼450 min. These parameters were chosen to mimic the experimental conditions of Dominguez et al. (2019 and under review).^12^ Before experiments, a phosphor screen (Kimball Physics model number PHOS-RP22SS-B5X5-R500) enabled determination of appropriate electron gun parameters, such as the focus for beam size and deflectors to center the beam on each sample (see Table 1). The experiments used an electron beam radius of ∼4 mm, but one experiment was performed with a smaller radius to determine how a change in surface area (SA), and thus higher electron beam intensity, could affect silicon oxidation (Table 1). The beam current was monitored every 10 min during each experiment to update fluence calculations using a retractable Faraday cup and picoammeter (Keithley 6485) to ensure that each sample received approximately the same electron fluence (= beam current × time). Images of the sample surface were also taken with a digital camera through a window in the vacuum chamber every 10 min to observe how the electron beam and surface changed throughout an experiment (Figure 2). At the end of each experiment, the sample was allowed to return to room temperature by turning off the cryostat, as previously described, and then removed from the UHV chamber for follow-up analyses.

**Figure 2:**
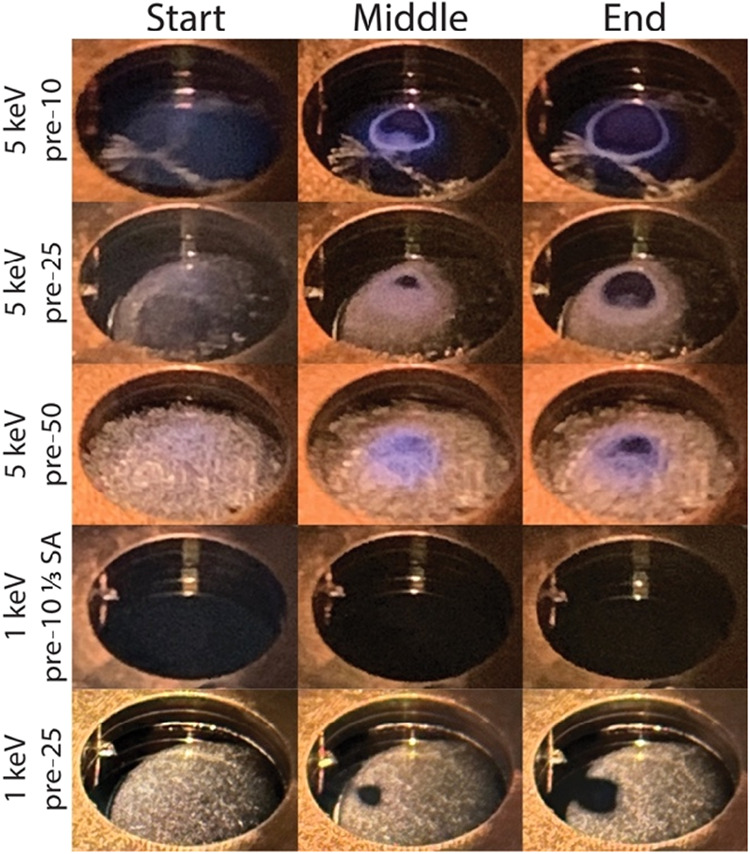
Photographs of sample surfaces at different time intervals during experiments while under UHV (columns = start, middle, end). Rows show different samples (*5 keV pre-10 μL, 5 keV pre-25 μL, 5 keV pre-50 μL, 1 keV pre-10 μL 1/3 SA*, and *1 keV pre-25 μL*). Only experiments with water-ice present before irradiation began (*pre-* samples) are included in order to observe how the beam affected the surface water-ice throughout the duration of an experiment.

### AFM-IR Measurements

Assessment of oxidation of the silicon substrates was made with point spectra and spectral mapping using AFM-IR (Bruker Anasys nanoIR3-s) at CSUSM. AFM-IR provides *in situ* nanometer spatial resolution of sample surface height variations and chemistry.^15,16^ This technique produces an infrared absorption spectrum by detecting a signal that is proportional to the thermal expansion force of a sample directly below an AFM tip.^17,18^ Data were collected using tapping mode and AFM tips with a resonant frequency of 75 ± 15 kHz (Anasys Instruments, model: PR-EX-TnIR-A-10). The IR source used is a pulsed Quantum Cascade Laser (QCL) with a spectral range of 905–1941 cm^–1^. Background spectra were taken across the spectral range, but sample point spectra were truncated to 915–1931 cm^–1^ to exclude noise at either end of the range. All spectra were collected at 4 cm^–1^ increments. Surface mapping was accomplished by moving the sample underneath the AFM tip via a computer-controlled stage to produce height and IR amplitude maps.

The AFM-IR was first optically aligned with a poly­(methyl methacrylate) (PMMA) standard; then, the IR laser was aligned and tuned to the PMMA. Point spectra of the PMMA were taken to confirm the instrument was optimized for sample analyses. IR laser alignment was stable once a tip was tuned, and backgrounds for point spectra analyses were collected at the beginning of each analysis day to account for possible environmental changes and power variation of the IR source. A Silicon Control was also run at the beginning of AFM-IR sessions as an additional confirmation of stable instrument optimization.

Samples were mounted on magnetic stainless-steel discs with carbon adhesive tabs, then magnetically mounted to the AFM sample holder. Different locations on the surface of each sample were examined to better understand oxidation heterogeneity. Line scans were taken at each location to produce height and IR amplitude maps. Scans were typically taken at a rate of 0.3–0.5 Hz, with a 200- to 500-point resolution for 0.3–30 μm^2^ regions, giving pixel sizes ranging from 0.6 to 150 nm. IR amplitude maps were run at 1100 cm^–1^, and in some cases 1150 cm^–1^, to highlight regions with possible Si–O bonds. Point spectra (1931–915 cm^–1^) were taken of the sample surface and particles of interest (if applicable) within each scanned region.

#### Analysis of AFM-IR Maps and Spectra

Surface maps and spectral data were processed using MATLAB. Height maps were flattened to account for any slight sample tilt by fitting each row to a first order polynomial (polyfit function) and subtracting the polynomial height from all of the data in that line. Map color bars were scaled using the maximum and minimum ranges of all locations for each sample. Point spectra were smoothed using the third order median filter function (medfilt1). Spectra were also scaled to the *Silicon Control* to normalize for amplitude variances across AFM sessions, which can be affected by AFM settings, including but not limited to the power, set point, IR focus, and laser pulse rate. Each spectrum was scaled to the *Silicon Control* spectra using the AFM-IR signal in the 1303–1931 cm^–1^ range, which is devoid of any Si–O peaks (1000–1200 cm^–1^),^14^ to normalize spectra from one AFM-IR session to another.

### SEM

#### Imaging

A selection of samples was chosen for additional imaging after AFM-IR data collection to explore surface textures at a larger scale (*Silicon Control*, *Water-Ice Only*, *5 keV pre-25 μL*, and *1 keV pre-25 μL*). Surfaces of samples were imaged with a 2 kV, 100 pA electron beam using two instruments: a dual beam focused ion beam—scanning electron microscope (FIB-SEM) (FEI Helios 660 FIB-SEM, University of Hawai‘i at Ma̅noa) and a scanning electron microscope (SEM) (ThermoFisher Varios 5UC EM, Molecular Foundry, Lawrence Berkeley Laboratory). Secondary electron (SE) images were collected using an Everhart-Thornley detector on both instruments.

#### EDS

The Helios FIB-SEM and the Varios SEM have silicon drift detector (SDD)-type energy dispersive spectroscopy (EDS) detectors (Oxford Instruments X-max N80 and EDAX Octane Elect, respectively). Spectra were quantified using background subtraction, peak fitting, and “remote standards standardless” quantitative analysis software (Oxford Instruments’ AZtec Energy Advanced Microanalysis System and EDAX’s APEX software, respectively) and normalized to 100%. Compositions are considered semiquantitative and used solely to look for the presence and/or absence of elements of interest.

### FIB and S/TEM

Representative surface locations for the *5 keV pre-25 μL* sample were selected for preparation using the Helios FIB-SEM of an electron transparent cross-section, hereafter called a FIB section. Standard FIB sample preparation techniques were used: C and Pt protective straps were deposited over the region of interest; the material on either side of the section was ion-milled away using 30 kV Ga^+^; the section was Pt-welded to a needle controlled by a micromanipulator, freed from the substrate, and Pt-welded to a Cu grid; ion milling at gradually decreasing beam current was used to thin the section to electron transparency; and final polishing with 5 kV Ga^+^ removed most of the ion beam-damaged surface layer (see Supporting Information for Figures S6 and S7).^19,20^ A fiber/rod on the surface of the *5 keV pre-25 μL* sample was also prepared as a FIB section. In order to have the best chance of retaining some of the fiber/rod, the FIB section was thinned to only ∼130 nm instead of the typical sub-100 nm target thickness. See Surface Particles section in Supporting Information for more details.

The FIB sections were imaged and analyzed at 300 kV and ∼250 pA electron beam currents with an FEI 60–300 low-base TitanX (scanning) transmission electron microscope (S/TEM, Molecular Foundry, Lawrence Livermore National Laboratory). High angle annular dark-field (HAADF) STEM and conventional dark-field (DF) and bright-field (BF) TEM images were collected. The TitanX S/TEM has a high solid angle (∼0.7 sr) energy dispersive spectrometer with four windowless X-ray SDDs (Bruker SuperX) for rapid element mapping and energy resolution of 140 eV at Mn Kα. Spectrum imaging was carried out by iteratively rastering the subnanometer electron probe across a region of interest while collecting X-ray fluorescence spectra at each pixel with a 10 eV/pixel dispersion for total acquisition times of ∼20–30 min, depending on map size. Element distributions are represented by color-coded maps generated from the integrated counts in energy windows around the characteristic K-edge X-ray energies for each element. EDS spectra were quantified using Esprit 2.3 software (Bruker Corporation) which uses factory-calibrated *k*-factors, background subtraction, peak fitting, and standardless quantification. Prior analysis of mineral standards of olivine, pyroxene, kamacite and pyrrhotite provided major element compositions with 1–2 atom % relative uncertainty and minor element compositions with 3–7 atom % relative uncertainty, depending on relative abundance.^21^

## Results

### Experimental Observations inside UHV Chamber

For the samples on which water was deposited before irradiation (named *pre-*), the ice on the sample surfaces showed various features (Figure 2; left column). The ice on the *5 keV pre-10 μL* sample (row 1 from the top in Figure 2) displayed vein-like features across the center and side of the surface. Alternatively, the *1 keV pre-10 μL* surface (row 4 from the top in Figure 2) did not visually show water-ice deposition, but IR peaks associated with Si–O bonds (described below in IR Point Spectra of Experiment Samples section) were observed for the sample surface with AFM-IR demonstrating that there was water-ice present during the electron irradiation experiment. The *5 and 1 keV pre-25 μL* samples (rows 2 and 5 from the top in Figure 2) showed a layer of ice that thinned toward the sample edges. Finally, the surface of the *5 keV pre-50 μL* sample (row 3 from the top in Figure 2) showed a thicker layer of ice that appeared to be a collection of ice crystals which spanned the entire surface of the sample.

During the experiments, visual changes were observed only for samples where water was deposited before electron irradiation began (*5 keV pre-10*, *-25, -50 μL, and 1 keV pre-25*, but not for *1 keV pre-10 μL 1/3 SA*). Photographs of the 5 keV irradiated samples showed a blue/purple glow on the surface of the sample that gradually formed a ring and increased in diameter throughout the duration of irradiation. This glow is likely caused by electron-bombardment-induced light emission, or electron-stimulated luminescence, of the water-ice.^22^ As the ring grew in size, the silicon surface below the ice appeared, suggesting the water-ice was sublimating over the course of these experiments. Additionally, when comparing the size of the ring for each sample at the same time intervals (Figure 2), we observed that the ring size was largest for the lowest volume of deposited water-ice, thus demonstrating a faster sublimation rate for thinner layers of water-ice. A concentrated region of water-ice dissipation was also observed for the *1 keV pre-25 μL* sample, but a purple/blue glow was not visually observed during that experiment.

### Post-experiment Sample Observations

Samples were imaged with the AFM-IR and SEM to observe surface visual and IR changes compared to the control samples. Most samples showed similar qualities, such as smooth surfaces with particles. However, some of the experimental samples showed unique textures that were not observed on the controls or *post-*irradiation experiment samples. We focus on these various features below.

#### Optical Images of 5 keV Pre-irradiation Samples

The unique textures of the 5 keV samples where water-ice was deposited before irradiation (*pre*-samples) were first observed via the optical images taken with the AFM’s optical microscope (Figure 3). The patterns on the *5 keV pre-10 μL* sample are the most pronounced with wavy alternating bands across the surface that highlight optically bright and dark features (Figure 3b). These patterns were observed near the center of the sample where the beam appeared to be the most concentrated (Figure 2, *5 keV pre-10 μL* middle and end). The 25 and 50 μL samples also showed patterns in the regions where the beam appeared the most intense (Figures 2 and 3c,d), but these samples showed patterns that looked speckled or as radiating rays, respectively.

**Figure 3:**
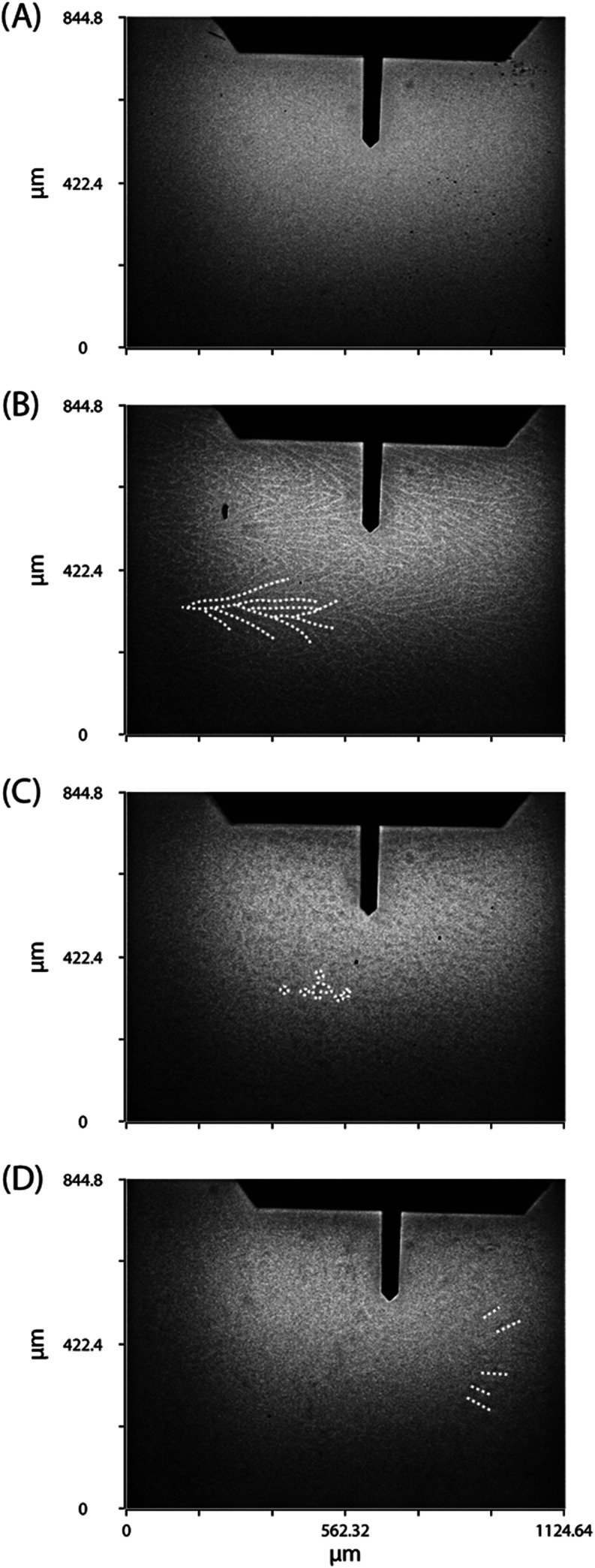
Representative AFM optical images for the surfaces of the (A) *Silicon Control*, (B) *5 keV pre-10 μL*, (C) *5 keV pre-25 μL*, and (D) *5 keV pre-50 μL* experiments. The images were edited to enhance the contrast, and white dashed lines were added to highlight surface textures for a portion of the observed areas (see Figure S2 in Supporting Information for original images). The *5 keV pre-10 μL* sample surface shows dendritic bands across the surface. The *5 keV pre-25 μL* sample shows a speckled or patchy surface. The *5 keV pre-50 μL* sample contains regions that appear to show a radial pattern. The *Silicon Control* does not exhibit a unique surface texture.

#### AFM-IR Maps

AFM-IR height and IR amplitude maps also revealed surface textures and particles at different locations across sample surfaces. Surface patterns were observed with AFM-IR mapping for the 5 keV experiments where water-ice was deposited before irradiation (*pre-10, 25*, and *50 μL*) (Figure 4). The type of surface texture is different for each sample, and the textures are concentrated near the most intense region of the beam (Figure 2). The *5 keV pre-10 μL* sample shows patterns that resemble branching rivers (Figure 4b) and the *5 keV pre-25 μL* sample patterns are shaped like islands (Figure 4c). The corresponding IR Amplitude maps also show these patterns (Figure 4b,c). The patterns in the *5 keV pre-10 μL* IR Amplitude map are very faint and may be due to map collection at 1150 cm^–1^ instead of 1100 cm^–1^, or likely due to less spectral signal across the sample causing surface variations to be more difficult to detect (see below in IR Point Spectra).

**Figure 4:**
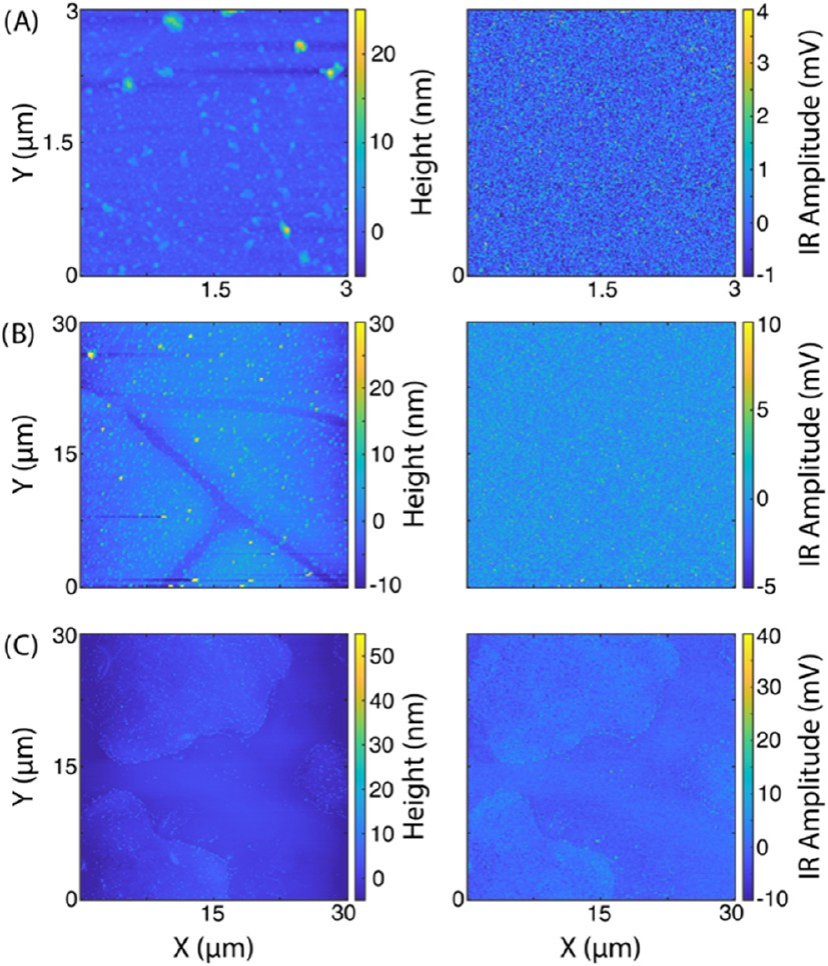
AFM-IR height and IR amplitude maps for the (A) *Silicon Control* (1150 cm^–1^ IR map) (B) *5 keV pre-10 μL* (1150 cm^–1^ IR map), and the (C) *5 keV pre-25 μL* (1100 cm^–1^ IR map) samples. The Height maps show examples of patterns for the surface texture of experimental samples that are not observed on the controls (*Silicon* and *Atmosphere Controls*, *Irradiation Only*, and *Water-Ice Only*). The corresponding IR Amplitude map for the *5 keV pre-25 μL* sample shows similar patterns, however, for the *5 keV pre-10 μL* sample the pattern in the IR is scarcely visible.

In addition to surface textures, particle-like surface features were observed on all samples, including controls. Only particles that peaked in the Si–O bond peak range were considered for this work and were only observed on the experimental samples with both water-ice and irradiation exposures. Other particles are likely contaminants remaining from the sample cutting and cleaning process. Distributions of particles (abundance, size, appearance) varied for each experimental sample and across individual sample surfaces. See Surface Particles section in Supporting Information for more details and examples of particles.

#### SEM Secondary Electron Images

Surface textures were further examined using SEM imaging after AFM-IR data collection (*Silicon Control*, *Water-Ice Only*, *5 keV pre-25 μL*, and *1 keV pre-25 μL*). Figure 5 shows the surface patterns (highlighted by the darker and lighter regions) of the four samples in greater detail with SEM and over a larger area compared to the AFM optical images (Figure 3). The *Silicon Control* sample shows scuff marks on the surface that likely result from the methanol cleaning process with Kimwipe tissues. The *Water-Ice Only* control shows a faint pattern on the surface, possibly due to residue from the evaporation of the water-ice as the sample came to room temperature under vacuum. The two experimental samples (*5 keV pre-25 μL* and *1 keV pre-25 μL*) show distinct patterns on their surfaces, reminiscent of a cantaloupe fruit rind. These textures were observed inside and partially outside of the hot spot locations observed during experiments, but not across entire sample surfaces (see Figures S3–S4 in Supporting Information for additional images). In comparison to the *Water-Ice Only* sample, the *pre-*irradiation experimental samples show well-defined patterns, demonstrating that the combination of water-ice and electron irradiation played a role in the creation of these patterns.

**Figure 5:**
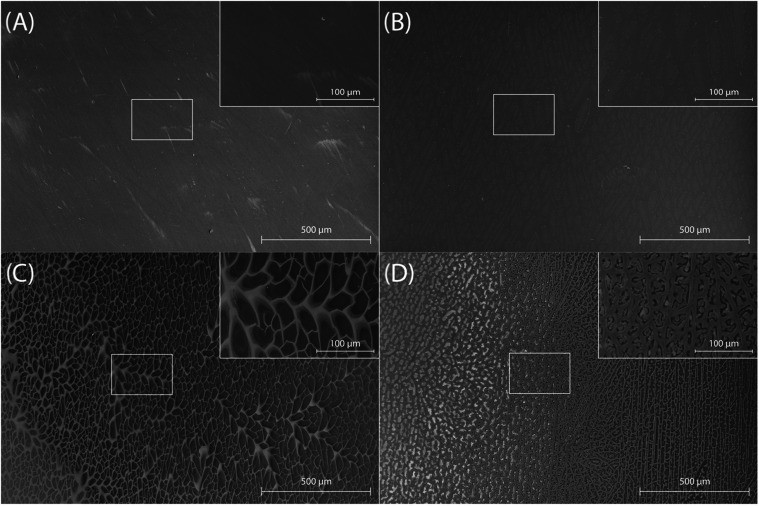
SEM SE images of silicon sample surfaces: (A) *Silicon Control*, (B) *Water-Ice Only*, (C) *1 keV pre-25 μL*, and (D) *5 keV pre-25 μL*. All images are 120× magnification with 750× magnification insets in the upper right corner (location of inset image indicated by the rectangle in the middle of each image). Each image highlights the surface textures of the samples: the control shows scuffs marks from cleaning with a Kimwipe tissue, the water-ice only sample shows faint evaporation patterns, and the 1 and 5 keV *pre-*irradiation experiment samples show well-defined textures resulting from the experiments. See Figures S3–S4 in Supporting Information for additional images.

### IR Point Spectra

Infrared point spectra were taken across the surfaces of each control and experimental silicon sample disk to understand how degree of oxidation due to electron irradiation varied with amount of water-ice deposition. The top of each sample was marked by scribing, and images were taken during the experiments so the location context could be tracked throughout data collection and analysis (Figures 2 and S1 in Supporting Information). We initially expected the electron beam to have uniform intensity over the sample surface and selected analysis areas at random. Further analysis revealed a beam hotspot, so locations were then more systematically selected to sample across the hotspot.

#### IR Point Spectra of Experiment Controls

IR spectra of control and experimental samples were collected to use as baseline comparisons and to provide a limit of oxide detection in the experimental samples (Figure 6). The SiO_2_ portion of the *Si/SiO*_2_ *Grating* shows the location of the dominant peak for Si–O bonds (∼1100 cm^–1^; Figure 6). The Si portion of the grating shows that there is no peak in the 1000–1200 cm^–1^ range and provides a standard spectrum of Si. The control silicon sample (*Silicon Control*; Figure 6) showed a very similar spectrum to the Si portion of the *Si/SiO*_2_ *Grating*. Silicon wafers rapidly develop a very thin native oxide on the surface with air exposure, and even though the samples were stored under vacuum with desiccant, the AFM-IR is likely not sensitive to such a thin oxide layer on the surface of the silicon samples. The silicon control was measured during different AFM-IR sessions and did not display signs of native oxide growth, thus providing a limit of detection for native oxide and indicates that the oxide observed for experimental samples is significant using our AFM-IR techniques. An additional control was stored in a sample box under ambient atmosphere and point spectra of this sample also did not show signs of native oxide growth (*Atmosphere Control*; Figure 6). Neither of the experimental controls, irradiated without water-ice (*Irradiation Only*) and only water-ice deposited but not irradiated (*Water-Ice Only*), showed evidence of Si–O bond formation, demonstrating that silicon oxidation is not caused by atmospheric interaction after a sample is taken out of the vacuum chamber.

**Figure 6:**
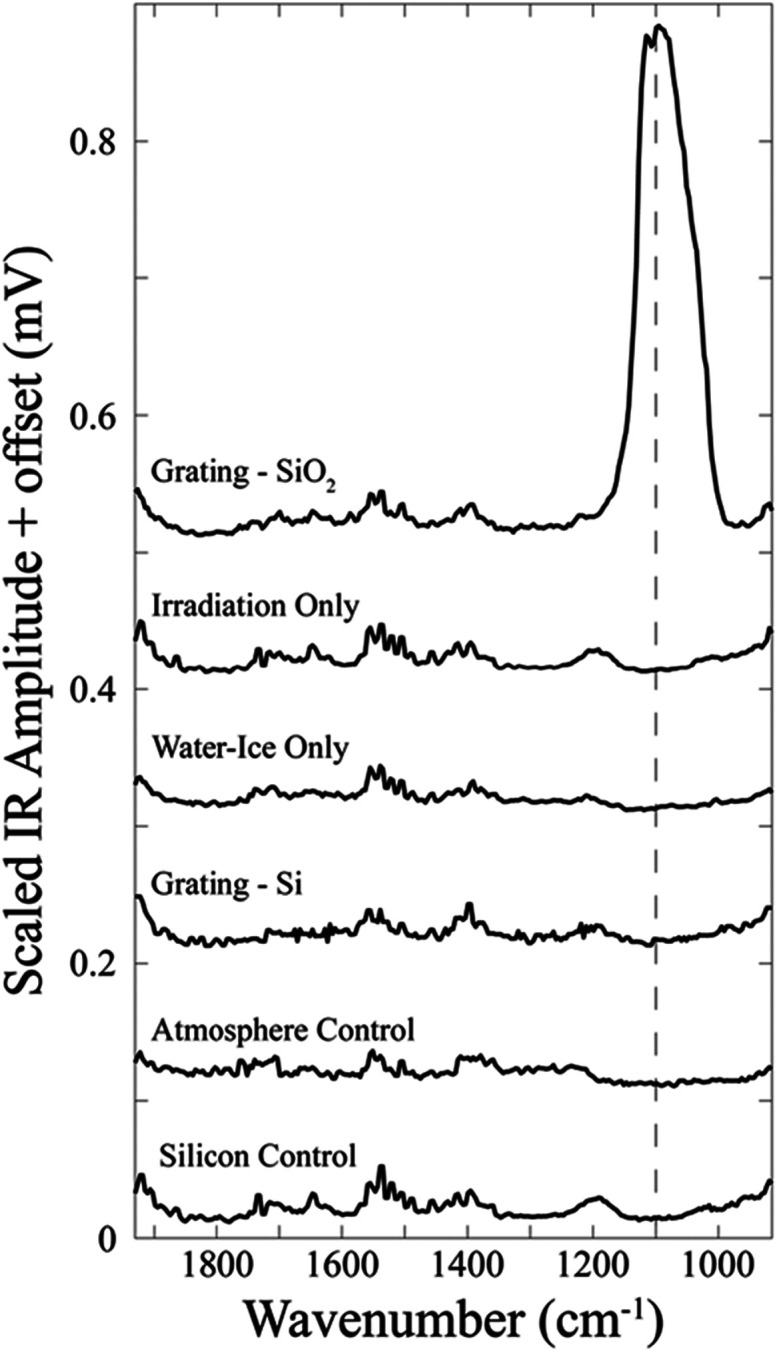
Point spectra of control samples and experiments (0.1 mV offset). The *SiO*_2_ *Grating* shows the dominant Si–O–Si bond peak at 1100 cm^–1^ that should arise from Si oxidation under experimental conditions. The control samples (*Silicon, Atmosphere, Irradiation Only*, and *Water-Ice Only*) did not show Si–O–Si bond peaks in the designated region (1200–1000 cm^–1^),^14^ demonstrating that native oxide was not detectable with AFM-IR, and that oxidation did not occur with irradiation or water alone under experimental conditions.

#### IR Point Spectra of Experiment Samples

IR point spectra of experimental samples show varying degrees of Si–O bond peak amplitudes within individual samples and in comparison to other experiments (Figure 7). Each panel displays groupings of spectra that are offset by 0.5 or 1.5 mV from the previous grouping for visibility. These groupings contain point spectra from a single experiment, and each spectrum is from a different location on that sample surface. Both panels are shown with the reference *Silicon Control* spectra (zero offset), demonstrating that the experiments only changed the silicon substrate spectra significantly in the 1000–1300 cm^–1^ range. This range is associated with Si–O–Si bonding, which indicates that additional Si–O bonds are generated compared to native oxide. Panel A of Figure 7 shows spectra from the surface of each sample with 0.5 mV shifts. Panel B shows particle spectra from each sample with 1.5 mV shifts because peak amplitudes of particle spectra are greater than surface peak amplitudes. Overall, each experimental sample shows elevated amplitudes around 1100 cm^–1^ (±10 cm^–1^), with some spectra having an additional peak around 1175 cm^–1^.

**Figure 7:**
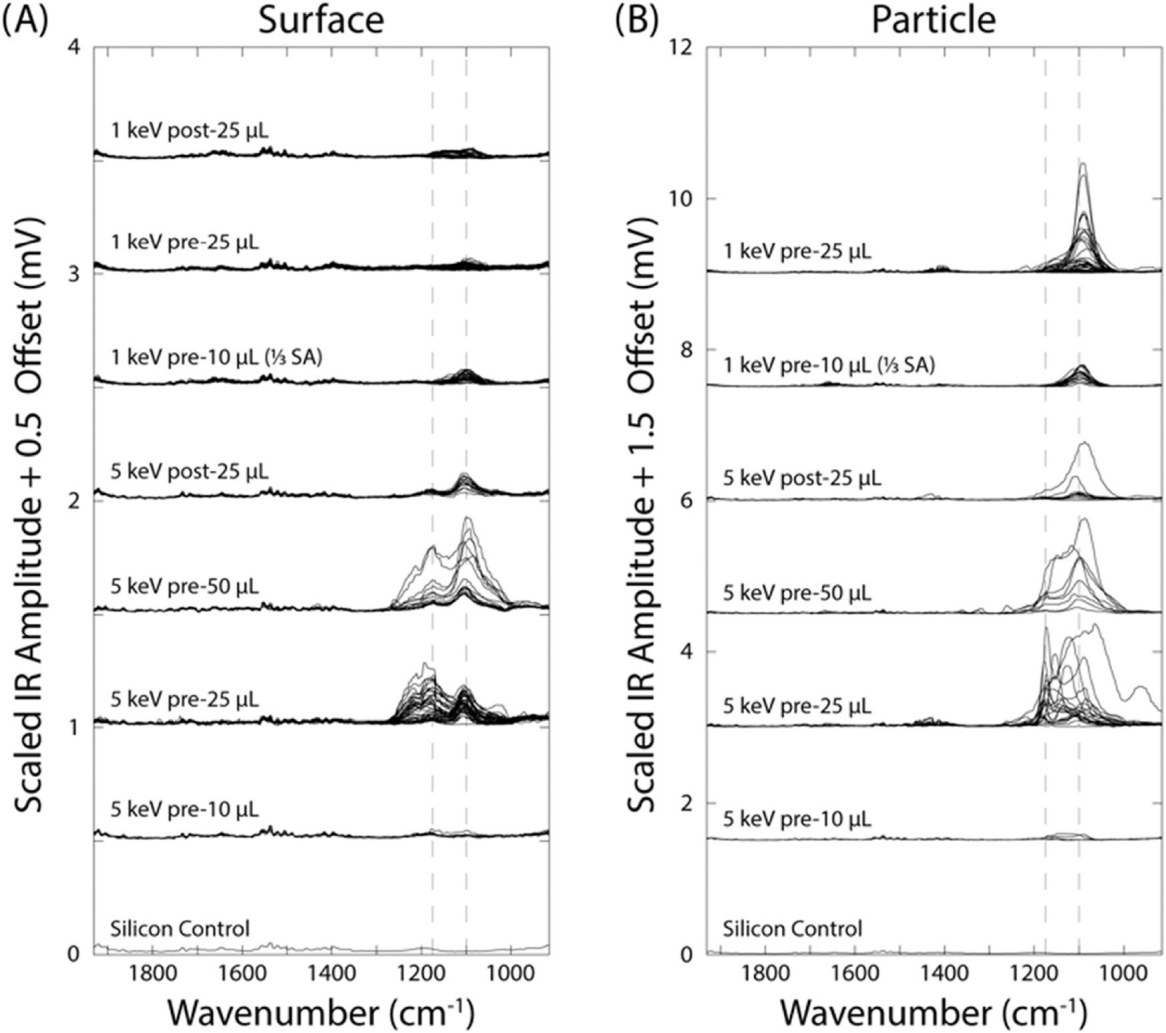
IR point spectra across each experiment sample. The *Silicon Control* spectrum used for scaling is shown at the bottom of each panel. Vertical dashed lines represent 1175 and 1100 cm^–1^. (A) Sample surface point spectra for each experiment with 0.5 mV shifts between experiments for visibility. (B) Particle point spectra for each experiment with 1.5 mV shifts between experiments. (The vertical mV shifts were set for each panel so the range and detail of surface and particle spectra could be observed.) Particles that peaked around 1100 cm^–1^ were not found in the scanned regions of the *1 keV post-25 μL* sample.

Variation in IR absorption amplitude was observed for each sample and across different experiments (Figures 7 and 8). The *5 keV pre-*irradiation experiment samples show an increase in peak amplitude as more water-ice is added to the sample surface, where the mean scaled maximum amplitude for the 10, 25, and 50 μL experiments were 0.03, 0.11, and 0.19 mV, respectively (Figure 8). A comparison of the *5 keV pre-25 μL* and *post-25 μL* experiments shows that the *pre-25 μL* spectra have greater amplitudes (0.11 and 0.08 mV mean scaled maximum amplitudes, respectively). The 1 keV experiments show a similar trend when comparing the *pre-* and *post-25 μL* samples, but overall, the 1 keV experiments have lower peak amplitudes compared to the 5 keV experiments. As for surface particles, their peak amplitudes are up to three times greater than the amplitudes associated with the broad regions of surface oxidation. While the detection of particles on experimental sample surfaces within the Si–O bond peak range suggests the experiments may play a role in their formation, determining their origin, whether experimentally produced or contamination, is outside the focus of the present work. Preliminary particle findings are detailed in Supporting Information.

**Figure 8:**
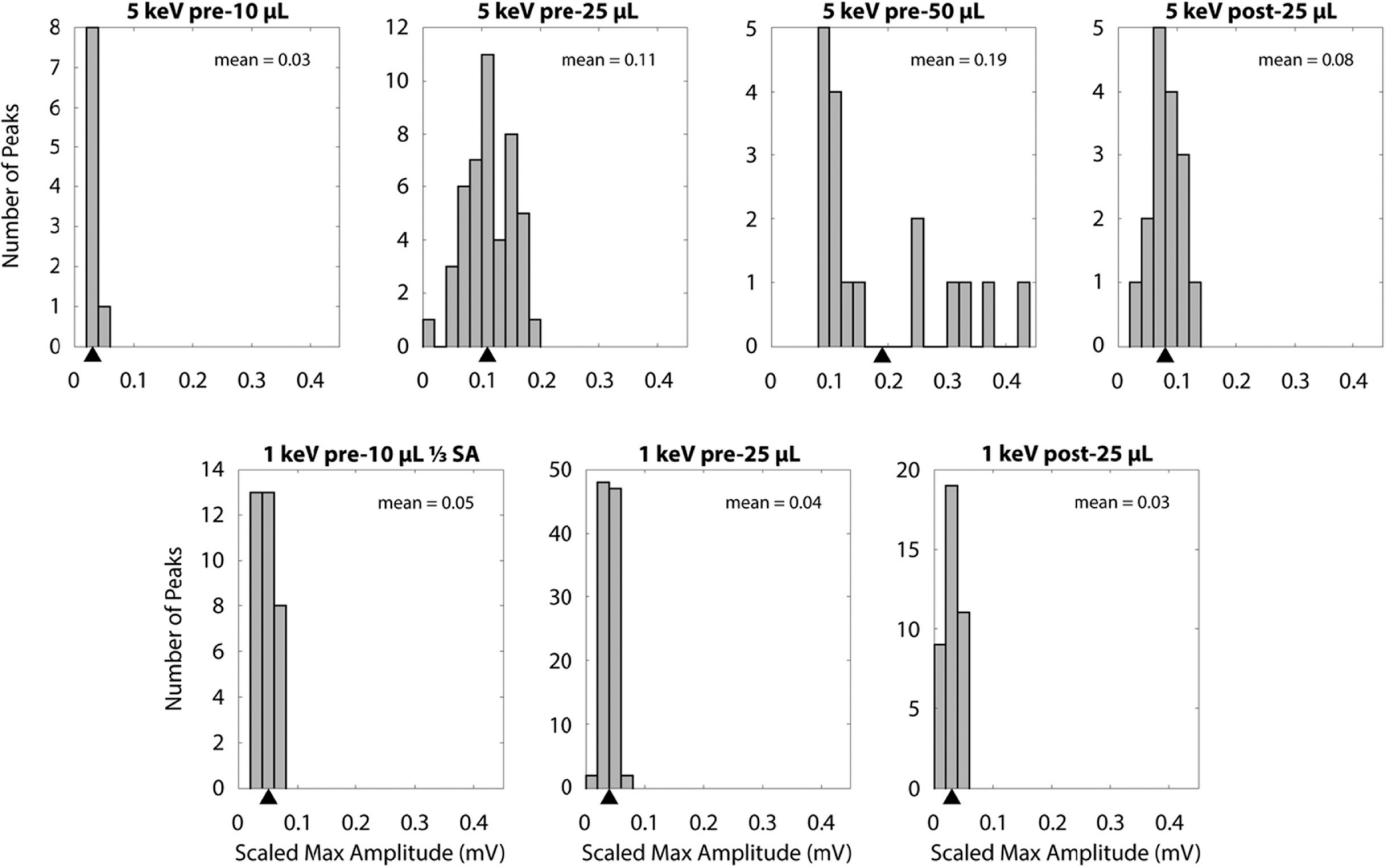
Histograms of scaled maximum amplitudes for each experiment sample. Black filled triangles on the *x*-axis of each experiment indicate the mean scaled maximum amplitude of the associated experiment.

#### Point Spectra Locations

In order to visualize the spectral variation across the surface of each sample, the average maximum intensity of the Si–O bond peak(s) at 1100 cm^–1^ (±10 cm^–1^) for each location was plotted on a circular representation of the sample surfaces, where the x- and y-coordinates were tracked with the AFM-IR software (Analysis Studio) (Figure 9). In the case of multiple spectra taken at a scan location, the maximum intensities were averaged for that location. In Figure 9a, each sample representation has a unique range with its own color bar, independent of the other samples, where dark red represents the highest amplitude on a sample surface. During beam parameter optimization with a phosphor screen, we observed that the electron beam profile was not uniform and a hot spot was observed (Figure S5 in Supporting Information). Optical imaging during experiments showed that the location of the glow and ice dispersion were consistent with the location of the hot spot on the phosphor screen (Figure 2). The best estimation of the beam hot spot location for each experiment is shown in Figure 9 with dashed circles, based on phosphor screen and optical imaging observations.

**Figure 9:**
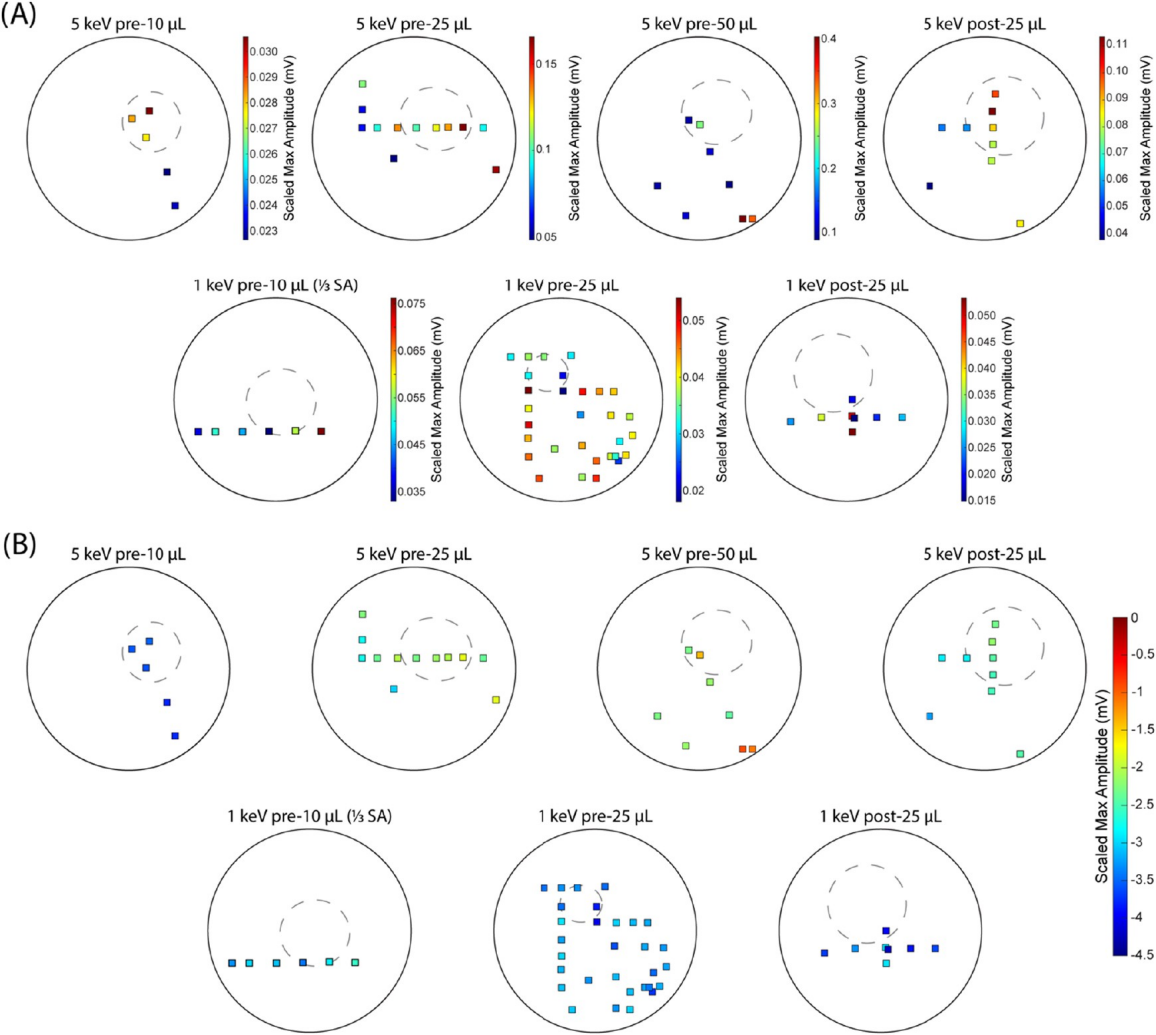
Locations of point spectra on the surface of each experiment sample (*D* = 12.4 mm). The color of each location marker represents the maximum Si–O bond peak amplitude around 1100 cm^–1^ (±10 cm^–1^). If multiple spectra were taken at a location, the color represents the average of the maximum amplitudes. Dashed circles represent where the beam was most intense, based on experimental images (Figure 2) and phosphor screen testing (Figure S5 in Supporting Information). (A) The color bar represents the maximum peak amplitude range per sample. Point spectra locations based on a sample’s individual scaling show the variability in peak amplitudes within each sample. (B) Plotting the natural log of these peak amplitudes and setting each sample to the same scale shows the variability within a single sample and allows comparison to the amplitudes of the other samples. While each sample shows internal variability, the 1 keV experiments show low amplitudes and little variation compared to the 5 keV experiments with pre-25 and 50 μL water ice deposition.

Some of the experiments in Figure 9a show a clustering of higher peak amplitudes (warmer tones) near the hot spot concentration, such as *5 keV pre-10 μL*, *5 keV pre-25 μL*, and *5 keV post-25 μL*, demonstrating the spectral effects of the hot spot observed in imaging (Figures 3–5). The remaining samples from both 5 and 1 keV experiments do not show apparent concentrations of high or low amplitudes across the sample surfaces. Figure 9b shows the natural log of the peak amplitudes in Figure 9a, and all samples are set to the same color bar scale. This data scaling shows the oxidation variation within a single sample and also demonstrates how the amplitude range of each sample compares to the others. Overall, the 5 keV experiments of post-25, pre-25, and pre-50 μL of water ice deposition showed the highest amplitudes with ∼2 to 6 times the mean scaled maximum amplitude of the remaining samples, which showed low amplitudes with little variation (Figures 7–9).

### S/TEM and SEM EDS of Experimental Surface Texture

The surface textures observed on multiple experimental samples (Figure 5) were examined further with SEM and S/TEM imaging and EDS techniques. A FIB section containing “on” and “off” texture surface material was extracted from the *5 keV pre-25 uL* sample (Figure S7). Comparing the “on” and “off” texture shows that both regions contain an oxide layer, however, the thickness of the “on” texture is approximately 2 times thicker than the “off” texture portion (Figure 10). SEM EDS analysis confirms that the “on” texture has a SiO_*x*_ composition (Figure S8).

**Figure 10:**
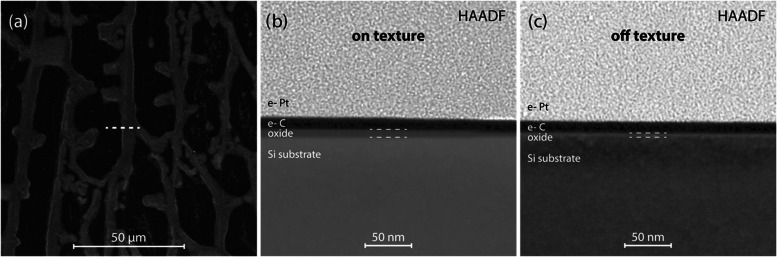
(A) SEM image of top-down view of the *5 keV pre-25 μL* sample surface showing the FIB section retrieval location with a dashed line. The left portion of the dashed line contains the “off” texture and the right side has the “on” texture portion. (B) S/TEM HAADF image of “on” texture and (C) “off” texture portions of FIB section, where the white dashed lines delineate the relative thicknesses of the oxide layers.

## Discussion

### Chemistry of Experimental Silicon Oxidation

The thermal energy in molecular cloud cores, where temperatures are generally 5–10 K, is extremely low (*k*_B_ *T* = 8.6 × 10^–4^ eV, for *T* = 10 K) and thus insufficient to break chemical bonds, which are on the order of *E*_b_ ∼ 5 eV. In contrast, energy deposition from cosmic rays, with energies on the order of *E* ∼ 10 GeV, would be sufficient to break a variety of chemical bonds. For example, the stopping power of a 40 MeV proton in silicon is 11.72 MeV cm^2^/g, so the amount of energy deposited into a small grain (*r* = 200 nm) is approximately 1090 eV.^23^ Consequently, even a single 5 keV electron, in our laboratory experiment, carries enough energy to break several thousand chemical bonds in Si (e.g., H–O (459 kJ/mol = 4.76 eV per bond), Si–Si (222 kJ/mol = 2.30 eV per bond), and Si–O (452 kJ/mol = 4.68 eV per bond)).^24^

Under the experimental conditions of this study, electrons impacting the sample surface have sufficient energy to break bonds in the water-ice layer and the silicon substrate. The resulting atomic and molecular fragments may recombine into their original compounds (Si–Si or H_2_O) or generate new Si–O bonds and other O and H bearing compounds. For example, oxygen atoms at 10 K may still migrate and react with other nearby molecules as suggested in prior studies^25^

1 $$O+O\to O_{2}$$

2 $$O_{2}+O\to O_{3}$$

Ozone (O_3_) in the gas phase can undergo mass independent fractionation (MIF), where the separation of isotopes does not depend on the masses of the isotopes.^26,27^ The experiments of Dominguez et al. (2019 and under review)^12^ suggest that MIF may occur in the solid-phase chemistry of O_3_.^12^ As a strong oxidant, O_3_ may react with unsatisfied Si- bonds (Si*) and potentially contribute to the observed oxygen isotope effects.

Electron irradiation of crystalline water ice has been shown to produce hydrogen and oxygen (H, O, H_2_, O_2_), as well as hydrogen peroxide (H_2_O_2_).^28–34^ Additionally, low-energy electron irradiation has been demonstrated to oxidize hydrogen-terminated silicon surfaces coated with water-ice layers.^35^ In our experiments, possible reactions include, but are not limited to

3 $$H_{2}O+e^{-}\to H+\mathrm{OH}$$

4 $$\mathrm{OH}+e^{-}\to O+H$$

5 $$O+{\mathrm{Si}}^{*}\to \mathrm{SiO}$$

or

6 $$\mathrm{OH}+{\mathrm{Si}}^{*}\to \mathrm{SiO}+H$$

7 $$H+H\to H_{2}$$

or

8 $$O+O\to O_{2}$$

9 $$O_{2}+{\mathrm{Si}}^{*}\to \mathrm{Si}O_{2}$$

or

10 $$O_{2}+O\to O_{3}$$

11 $${\mathrm{Si}}^{*}+O_{3}\to \mathrm{SiO}+O_{2}$$

or if there is a Si atom on the surface with two unpaired electrons (*Si*)

12 $${\;}^{*}\mathrm{Si}{\;}^{*}+O\to {\;}^{*}\mathrm{SiO}$$

13 $${\;}^{*}\mathrm{SiO}+O\to {\mathrm{SiO}}_{2}$$

Other arrangements of O (O_2_, O_3_, and OH) are also possible.

We also consider the possibility that the surface oxidation observed is due to thermal oxidation, like that used in semiconductor processing to oxidize silicon wafer surfaces under “wet” conditions. However, the surface temperature of the experiments under our conditions are much lower than those typically used for efficient thermal oxidation (800–1200 °C).^36^ While the temperature gauge in our experiments measures only the edge of each 12.4 mm diameter sample, the temperature at the beam hotspot is not expected to differ by many hundreds of Kelvin without registering on our temperature gauge or resulting in sample breakage. Additionally, increased oxidation relative to the controls is observed for the *post* experiments where the beam is turned off after irradiation and the sample remains cold during water deposition. Therefore, our experimental conditions demonstrate that bond breakage and reforming via residual energy or vacancies plays a key role in oxidation of the silicon surface.

### Effects of Experimental Conditions on the Creation of Si–O Bond Peaks

AFM-IR point spectra taken across the surface of each sample revealed how the experimental conditions affected the amplitude of the Si–O bond peak in relation to their location on a sample. The lack of Si–O bond peaks observed for the control experiments (*Irradiation-* and *Water-Only*) demonstrates that Si–O bonds were formed when electron irradiation and water-ice coexisted within the experimental time frame (Figure 6). Additionally, the lack of Si–O bond peaks for the control experiments demonstrates that the amount of oxide formed from the experiments is greater than typical native oxide abundances. Experimental Si–O bonds peaks were observed around 1100 cm^–1^ (±10 cm^–1^) and in some cases near 1175 cm^–1^, both of which are in the expected range of 1200–1000 cm^–1^ for Si–O bond peaks (Figure 7).^14^ The additional peak around 1175 cm^–1^ observed on some surfaces and particles may suggest the formation of a quartz polymorph (e.g., α-quartz), but additional characteristic IR peaks needed for reliable classification are outside of the spectral range of the CSUSM AFM-IR.^13^

Electron irradiation experiments were conducted at two energies (1 and 5 keV) to examine how electron energy influences visual and spectral responses of the samples. Experiments performed at 5 keV with water ice deposited before irradiation (*pre-* experiment samples) exhibited a circular glow. The glow appeared brightest at the interface between the remaining water-ice and the exposed silicon substrate, which corresponds with the beam hot spot (Figure 2). In contrast, no visible glow was observed on the *1 keV pre-*experiment samples. This absence indicates that a higher threshold of energy deposition and ice thickness is needed to produce a visually detectable glow, rather than suggesting no glow occurs under these conditions.

This observed glow is consistent with findings by Gudipati et al.,^22^ where a variety of ices were electron irradiated to understand their emission characteristics for determining the chemical composition of Europa’s surface during future missions. They attributed the glow to a variety of phenomenon, such as but not limited to, the excited to ground-state transition emission in water molecules and the electron-stimulated luminescence of OH radicals created in the ice during irradiation.^37,38^ Although Gudipati et al.^22^ used thicker ice layers, higher energies, and shorter irradiation durations, their average flux (∼1 × 10^12^ electrons cm^–2^ s^–1^) was lower than that used in our experiments (∼1 × 10^14^ electrons cm^–2^ s^–1^; Table 1).

Comparisons between the 5 and 1 keV experiments showed that while oxidation occurs with 1 keV electron irradiation, the Si–O bond peak intensities are notably less prominent compared to the 5 keV experiments (Figure 7). Electron irradiation simulations (CASINO^39^) show that for a given ice layer thickness, 5 keV electrons penetrate deeper into a Si sample compared to 1 keV electrons, supporting the difference in peak intensity amplitudes for these energy conditions (Figure S9 in Supporting Information). Furthermore, Figure 9b, which normalizes emission amplitude between the two energy regimes, illustrates that the amplitudes, and therefore the oxidation, in 1 keV experiments is significantly reduced relative to the 5 keV experiments (Figure 9b). The limited variation in oxidation signal across the 1 keV samples makes it difficult to assess the degree of heterogeneity.

The effect of water-ice abundance is best observed in the 5 keV experiments, where the maximum Si–O amplitude measured on the sample surface scales with increasing water-ice deposition (Figure 7). Even though the same protocol was used to deposit water in the different water-ice dosage experiments, there are visual differences in the distribution of water ice on the surfaces, including shadowing due to the lip of the holder (Figure 2). The most obvious of these differences is the systematic increase in water ice thickness as the amount of water injected increased, which correlates with greater Si–O peak amplitudes around 1100 cm^–1^ (Figures 7–9). Future experiments would benefit from improving the setup to ensure even water-ice deposition, as well as adding a method to quantify the amount of water on each sample surface, for example a quartz crystal microbalance.

The timing of water-ice deposition before (*pre-*) or after (*post-*) electron irradiation also affects the peak amplitudes measured (Figure 7). The amplitude difference between the *5 keV post-* and *pre-25 μL* experiments shows that electron irradiation of an iceless surface followed by water-ice deposition will still oxidize the silicon surface, but the oxidation will occur at a lower efficiency compared to simultaneous water-ice and irradiation conditions. The difference in peak amplitudes for *pre-* versus *post*-irradiation experiments may be due to the continual breaking and reforming of bonds when water-ice is present during irradiation, versus the *post*-irradiation experiment where newly introduced water-ice molecules will only interact with defects available after irradiation ends.^40,41^ These observations strongly suggest that the lifetime of Si lattice defects under these conditions is longer than the time that elapsed between the end of irradiation and the beginning of water ice deposition (∼30 min to first water-ice deposition). These defects are likely still available due to a lack of nearby atoms and the minimized movement of atoms at these cold experimental temperatures. Additionally, quantum tunneling of oxygen atoms generated through the dissociation of water dominates up to 20 K and may play a role in the observed silicon oxidation.^42^ Future experiments could probe the time and conditions necessary for defects to anneal and how temperature may play a role in the annealing rate.

The effect of beam size on the formation of Si–O bond peaks was also explored for experiments conducted at 1 keV. The beam radius experiment, *1 keV pre-10 μL 1/3 SA* (Table 1), exposed a third the surface area (SA) to electron irradiation relative to the normal experiments. This experiment produced a larger peak amplitude around 1100 cm^–1^ compared to the *1 keV pre-25 μL* and the *5 keV pre-10 μL* experiments (Figure 7), suggesting that higher beam intensities result in greater concentrations of oxidation of silicon surfaces.

### Surface Heterogeneity

The spatial heterogeneity of surface oxidation of an experimental sample is reflected in the range and distribution of peak Si–O bond amplitudes around 1100 cm^–1^ (±10 cm^–1^) (Figures 7–9), which we attribute to variations in electron beam energy distribution and variable water-ice thicknesses.

We observed higher AFM-IR absorption at 1100 cm^–1^ in regions of the sample surfaces that aligned with the hot spot location of the beam (see Figure 9). This effect is best seen in the *5 keV post-25 μL* sample experiment, where irradiation happened in the absence of a deposited water-ice layer (Figure 9). A similar concentration of higher amplitudes is observed on the *5 keV pre-10 μL* and *5 keV pre-25 μL* samples, although not to the same degree as the *5 keV post-25 μL* sample. No obvious hotspots are observed spectrally for the remaining samples, especially for the 1 keV experiments, which is likely due to the lower range of peak amplitudes produced by a lower electron energy (Figure 7).

The well-defined oxidation hotspot observed on the *5 keV post-25 μL* sample is likely due to the absence of variable water-ice thicknesses shielding electrons from reaching the silicon surface.

In the case of the *pre*-experiment samples where water-ice is deposited before electron irradiation, the icy layer on the silicon surface appears uneven in optical photographs (Figure 2). These variations in water-ice thickness likely caused variations in the scattering and production of secondary electrons that reached the silicon surface, leading to variations in energy deposition. CASINO simulations show that water-ice thickness variation has a strong effect on the electron intensity reaching the silicon surface (Figure S10).^39^ This small-scale heterogeneity of ice layer thickness and beam intensity variations are likely key contributors to the range of Si–O peak amplitudes observed in this study.

When observed using AFM-IR and SEM, samples that were exposed to water-ice and irradiation exhibited surface textures, while the control samples did not (Figure 5). The *5 keV pre-25 μL* sample surface showed a denser surface texture pattern, suggesting that a higher fraction of the surface was damaged, compared to the *1 keV pre-25 μL* sample (Figure 5c,d). Additionally, the observation that the *5 keV pre-25 μL* sample has higher Si–O peak amplitudes compared to the *1 keV pre-25 μL* sample strongly suggests that the denser texture is the result of having higher amounts of oxidation on the surface. The correlation between surface texture and oxidation was further confirmed via FIB section analysis of the *5 keV pre-25 μL* sample surface (Figure 10). Here, we directly confirmed oxidation in a region that had surface texture (“on” texture) compared to a neighboring region (“off” texture) and found that the textured region had a thicker (∼2×) oxide layer, where the composition was confirmed to be SiO_*x*_ using EDS (Figures 10 and S8).

Surface textures in our experiments are similar to the “tree” structured surface textures (long radiating branches) observed in dielectric breakdown experiments performed to simulate lunar space weathering.^43,44^ One such set of experiments observed discharge phenomena on silicate minerals with varying Fe contents at room temperature with 30 kV electrons and fluxes ranging from 10^11^ to 10^13^ electrons cm^–2^ s^–1^.^44^ The visual similarities are suggestive of a similar phenomenon occurring on the ice covered surfaces (Figures S3–S4). Additional experiments, perhaps with other volatile ices, could help clarify this potential connection.

### Experiment Implications

Our results show that keV electron irradiation renders refractory solid surfaces chemically reactive even at ∼10 K. Both concurrent (pre-) and nonconcurrent (post-) water-ice deposition experimental samples demonstrate that irradiation-induced surface modifications occur and, in the case of the *post*-experimental samples, persist beyond active electron irradiation exposure while under astrophysical conditions. These findings are consistent with Dominguez et al. (2019 and under review),^12^ and suggest that electron-induced defects (e.g., dangling bonds and vacancies) interacting with condensed volatiles may lead to changes in the isotopic composition of metal-oxides. We expect this behavior to extend to a wider range of refractory materials and volatile species. The degree of this behavior may also differ if the material is crystalline or amorphous.

In astrophysical and planetary environments, energetic particles (e.g., cosmic rays) generate secondary electrons that continuously irradiate surfaces such as interstellar dust grains, regolith in permanently shadowed regions on the Moon and Mercury, and asteroids. Our experiments indicate that such irradiation, in the presence of volatile ices, can drive chemical modification even where thermal reactions are suppressed, with important implications for the chemical and isotopic evolution of cold planetary materials.

Isotopic exchange arises from irradiation-induced bond scission and defect formation, which disrupt local bonding (e.g., Si–O–Si) and lower barriers to rearrangement. Interaction with H_2_O ice or its dissociation products enables reformation of metal–oxygen bonds incorporating oxygen from the ice, providing a pathway for solid–volatile isotopic exchange even at cryogenic temperatures. The exchange in *post*-irradiation experiments demonstrates the persistence of defects, while the extent of isotopic modification is expected to depend on defect density, irradiation fluence, and the isotopic composition of the volatile reservoir.

## Conclusion

We found that exposure to electron irradiation and water-ice coated silicon results in the oxidation of silicon, as shown by AFM-IR and electron microscopy techniques. Oxidation was observed under a variety of experimental conditions, with the amount of water-ice and energy deposition being the most influential. Interestingly, we observed oxidation in samples that were irradiated prior to their exposure to water-ice (*post*-samples), which suggests that irradiation-induced defects in the Si surface leave the surface susceptible to subsequent oxidation by water-ice. Variations of the Si–O IR absorption signal within a sample are attributed to heterogeneities in energy deposition and water-ice thickness. Optical and SEM imaging of surfaces revealed surface textures that correlated with electron energy and exposure to water-ice deposition. Our work shows that solid surfaces in cold astrophysical environments are susceptible to chemical and isotopic changes.

## Supplementary Material



## Notes

Additional images (experimental, AFM optical, and SEM), electron beam hot spot determination, and details for surface particle examples (PDF)
