# Supplementary material for: Detection of Simulated Space Weathering on Electron Irradiated Water-Ice Coated Silicon Using AFM-IR, SEM, and S/TEM Techniques
Source: ACS Earth Space Chem. 2026 Jun 27;10(7):1817–30. doi: 10.1021/acsearthspacechem.6c00135 (PMC13383826; doi:10.1021/acsearthspacechem.6c00135)
Supplement: Supplementary file 1 [file sp6c00135_si_001.pdf]

Supplementary Materials for

Detection of Simulated Space Weathering on Electron Irradiated Water-Ice Coated Silicon Using AFM-IR, SEM, and S/TEM Techniques

Caroline E. Caplan [a*], Hope A. Ishii [b], Jeffrey J. Gillis [c], Kevin D. McKeegan [d], Ming-Chang Liu [d], and Gerardo Dominguez [a*]

[a] *Physics Department, California State University, San Marcos, CA 92096, United States*
[b] *Hawai'i Institute of Geophysics and Planetology, University of Hawai'i Mānoa, Honolulu, HI 96822, United States*
[c] *Department of Physics, Washington University in St. Louis, St. Louis, MO 63130, United States*
[d] *Department of Earth, Planetary, and Space Sciences, University of California, Los Angeles, CA 90095, United States*
*Email: ccaplan@csusm.edu & gdominguez@csusm.edu

This PDF file includes:

Figures: Figs. S1 to S13

Section: Surface Particles

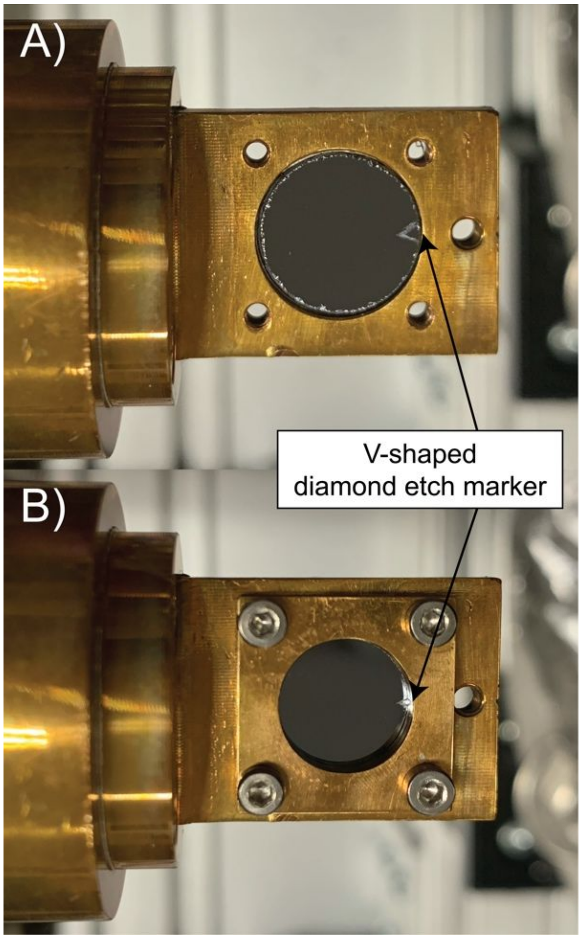

Figure S1. Representative silicon disk in the cryostat holder A) without and B) with the bolted cover. A V-shaped marker was diamond etched at the top of each sample (right side of sample here) to keep track of the sample positioning during experiments and analysis.

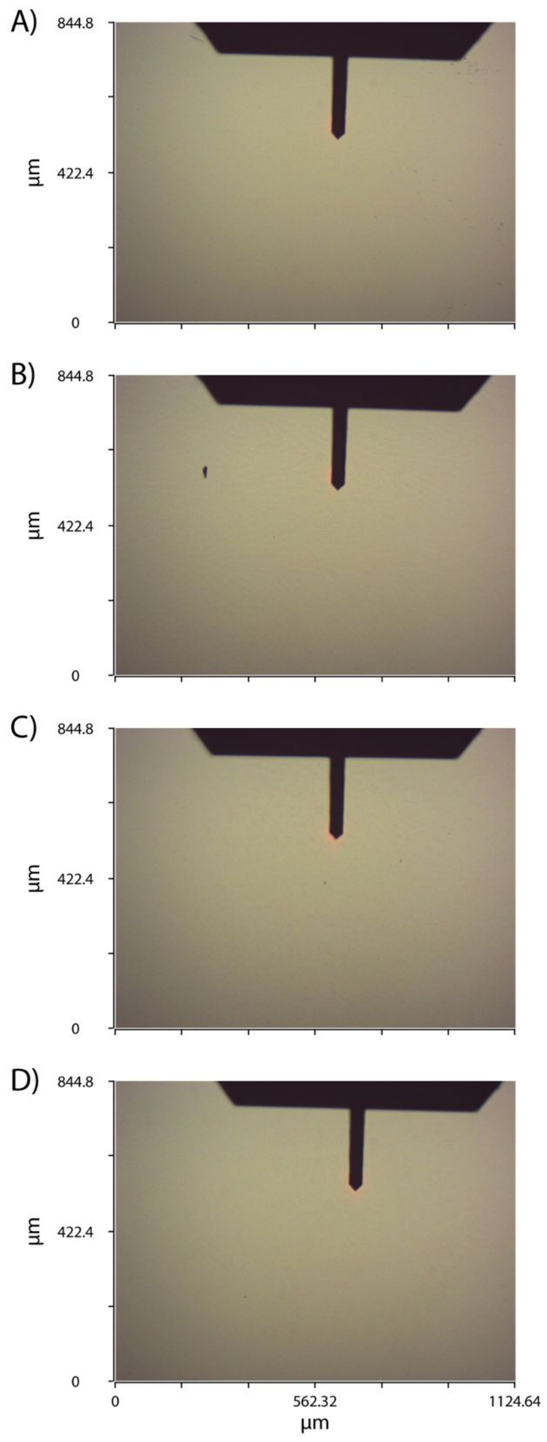

Figure S2. Representative AFM optical images for the surfaces of the A) *Silicon Standard*, B) *5 keV pre-10 µL*, C) *5 keV pre-25 µL*, and D) *5 keV pre-50 µL* experiments. These images are the original and unedited versions of Fig. 3 in the manuscript.

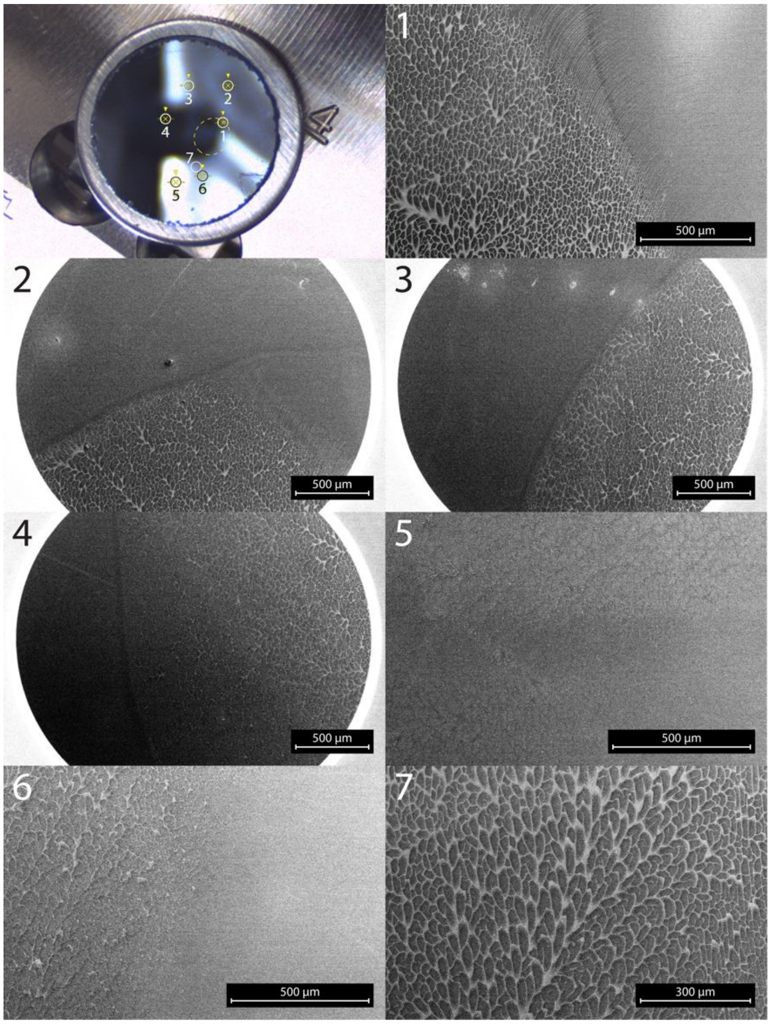

Figure S3. SEM imaging of the *1 keV pre-25 μL* sample surface. The top left is the NavCam image of the sample in the SEM and is marked with locations for the corresponding numbered images. The V-shaped marker is visible at the lower right, similar to Fig. S1. The dashed oval is the same as Fig. 9 for the *1 keV pre-25 μL* (water deposited before irradiation) sample but is rotated due to the orientation of the sample in the SEM, and represents the hot spot seen on the sample surface during the experiment (Fig. 2).

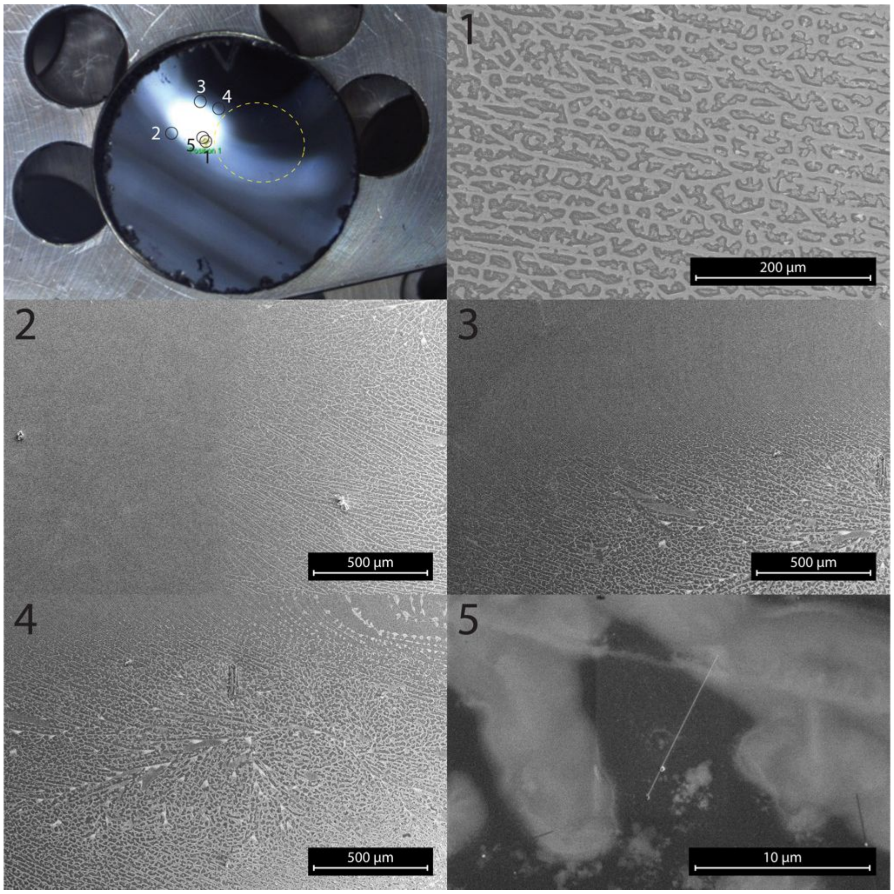

Figure S4. SEM imaging of the *5 keV pre-25 μL* sample surface. The top left is the NavCam image of the sample in the SEM and is marked with locations for the corresponding numbered images. The dashed oval is the same as Fig. 9 for the *5 keV pre-25 μL* sample and represents the hot spot seen on the sample surface during the experiment (Fig. 2). The top of the sample is identified by the V-shaped mark, similar to Fig. S1. The fiber/rod on the surface of location 5 was FIB sectioned for S/TEM analyses (Fig. S6).

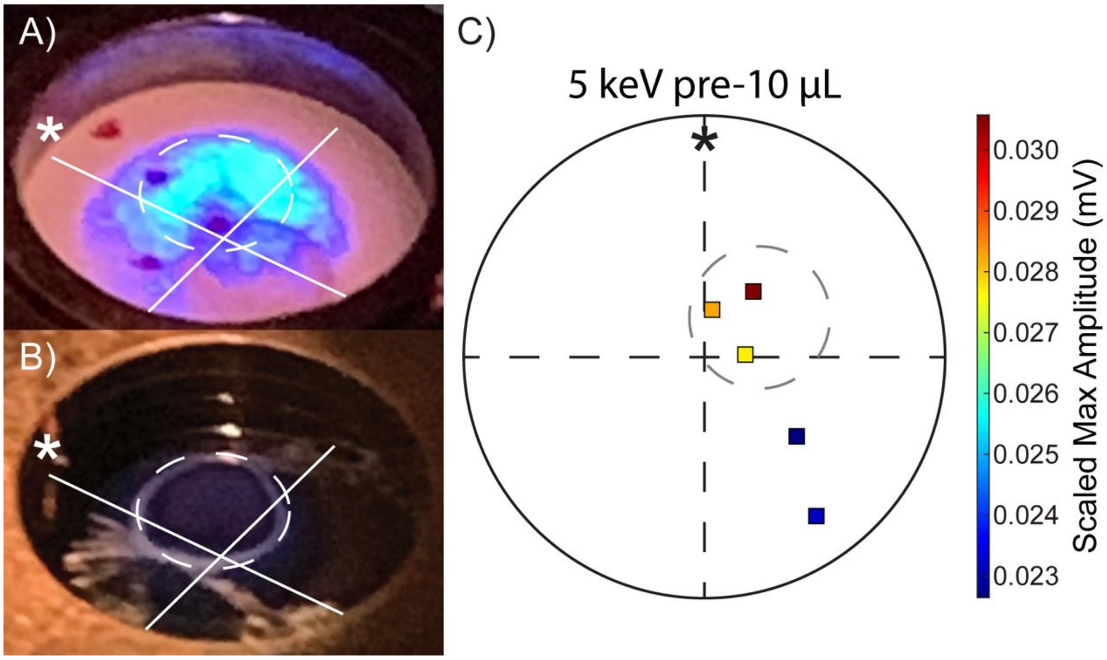

Figure S5. Example comparison of the electron beam hot spot on A) the phosphor screen (red dots are ~2 mm apart), B) the *5 keV pre-10 μL* sample at the end of the experiment, and C) a schematic of the sample surface (D = 12.4 mm) with locations of AFM-IR point spectra with a color bar showing the scaled max amplitude around 1100 cm^{-1} of that location (see Fig. 9). The asterisks (*) represent the top of the sample where the V-shaped marker is located (see Fig. S1). The patchy circle at the bottom of the phosphor screen (A) is an old burn mark from testing at higher currents.

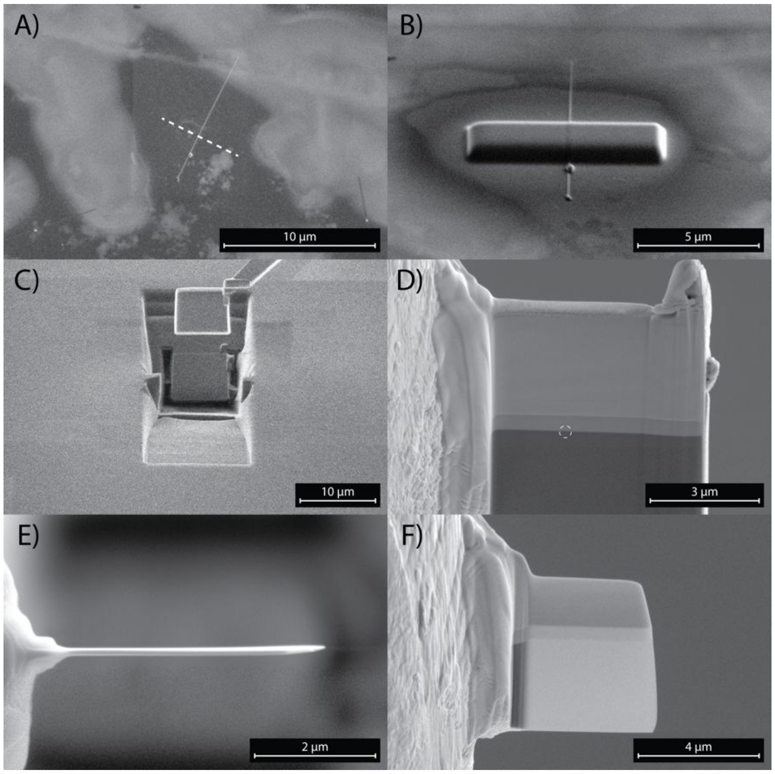

Figure S6. SE images of FIB section preparation for a fiber/rod on the surface of the *5 keV pre-25 μL* sample. A) Top-down view of fiber/rod on sample surface, where white dashed line represents location of FIB section extraction, and B) side view of fiber/rod with a protective Pt strap. C) Ion-milled trenches on either side of the Pt strap with section lifted out after cutting free from the surface. D) FIB section welded to copper grid and cleaned with fiber/rod location marked by a white dashed circle. E) Top-down and F) side view of thinned section for S/TEM analyses.

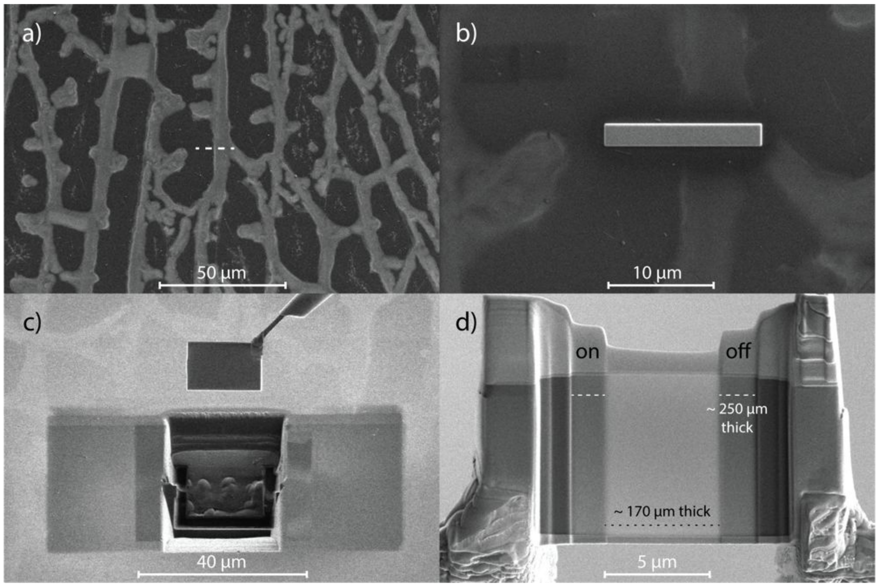

Figure S7. FIB section preparation for an on and off textured surface of the *5 keV pre-25 μL* sample. A) SE image of top-down view of sample surface (dashed line is retrieval location) and B) closer view with a protective Pt strap. The left portion of the Pt strap is covering the "off" texture portion and the right side is the "on" texture portion. C) Ion-milled trenches on either side of the Pt strap with section lifted out after cutting free from the surface. D) FIB section welded to copper grid at bottom left and right corners. The section was thinned to different thicknesses (~250 and ~170 μm) for analysis. This orientation shows the "on" texture on the left side and the "off" texture is on the right side.

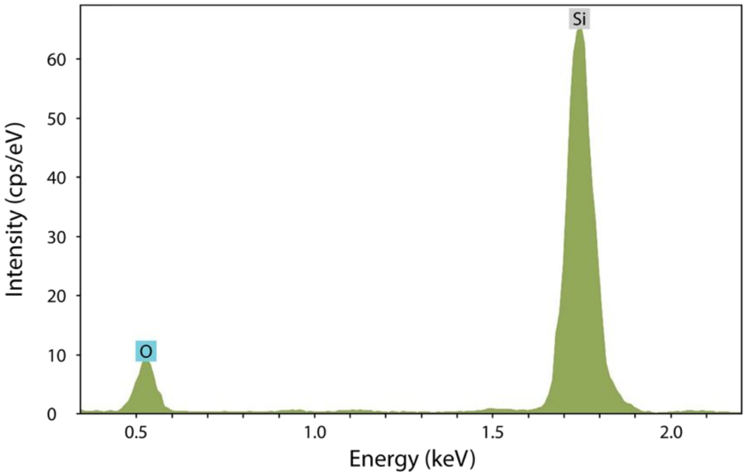

Figure S8. SEM EDS spectra of the "on" texture surface of the *5 keV pre-25 μL* sample in Fig. S7 shows a SiO_x composition.

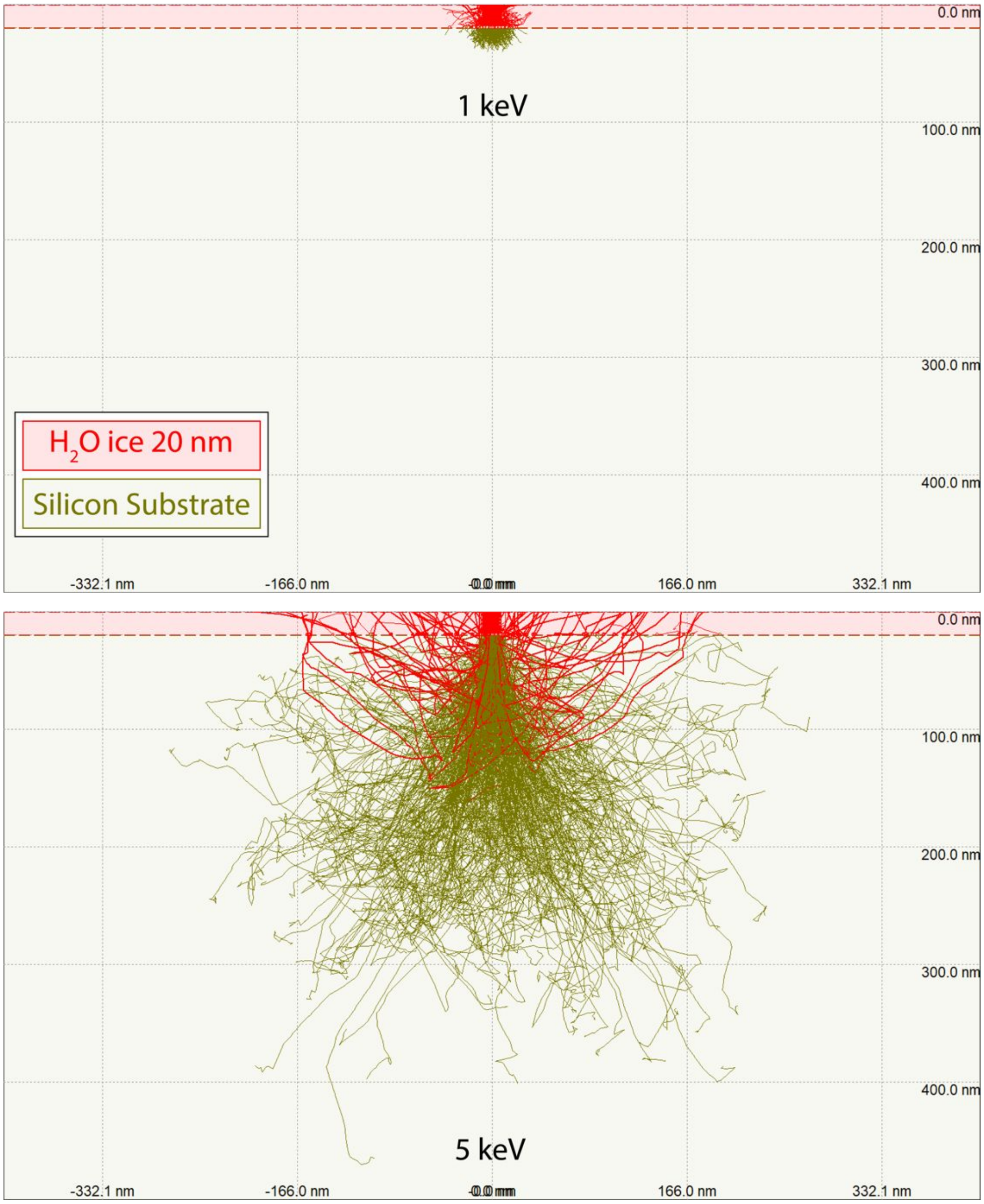

Figure S9. CASINO simulations for a 10 nm beam of 1 keV (top) and 5 keV (bottom) electrons (n=500) irradiating a 20 nm thick layer of water-ice on a silicon substrate.

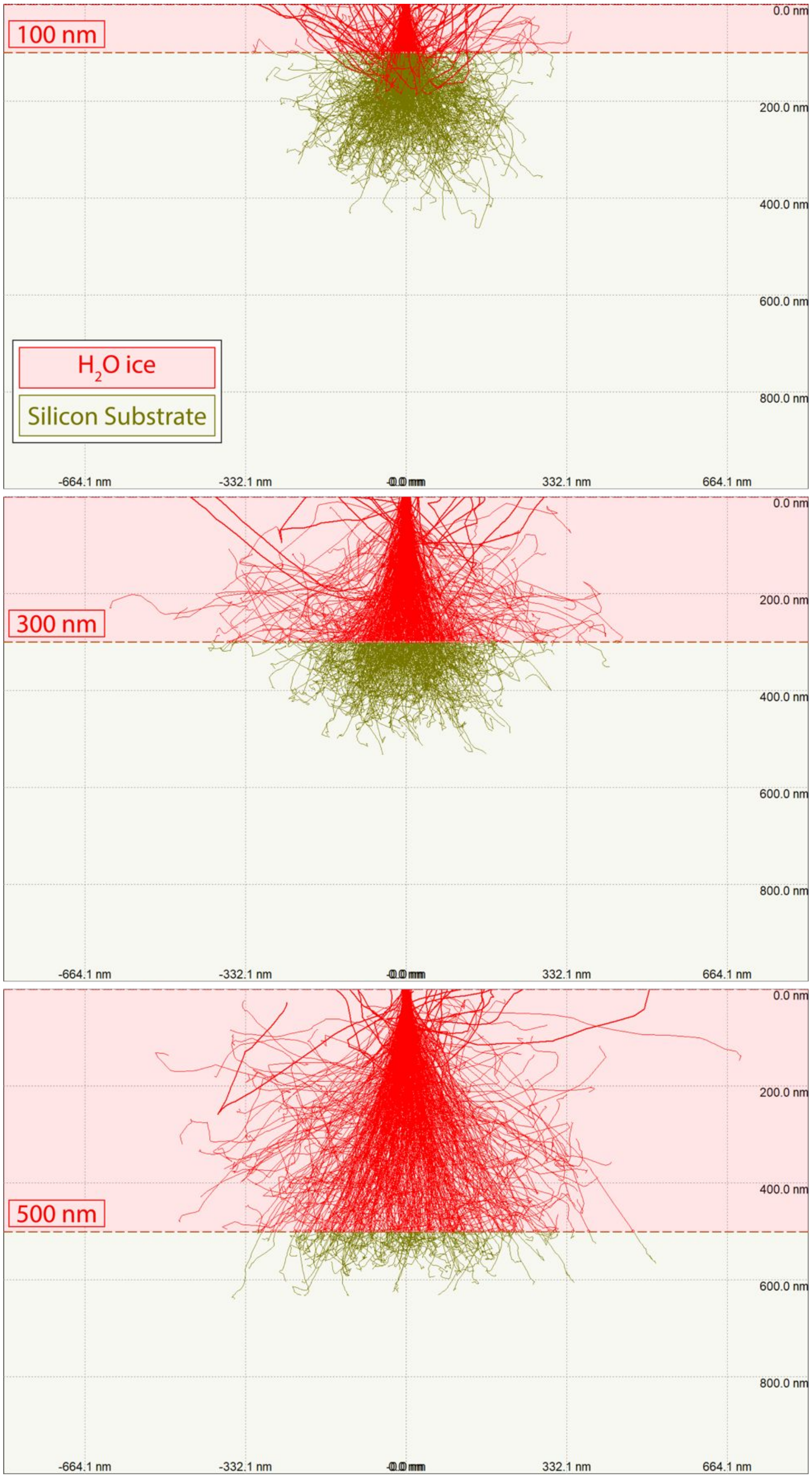

Figure S10. CASINO simulations for a 10 nm beam of 5 keV electrons (n=500) irradiating a 100 nm (top), 300 nm (middle), and 500 nm (bottom) thick layer of water-ice on a silicon substrate.

SURFACE PARTICLES

AFM-IR of Surface Particles

Detailed AFM-IR maps of samples revealed the presence of surface particles with an Si-O bond peak indicating they may have formed during the experiments (higher amplitudes in 1100 cm^{-1} IR maps; Fig. S11). Particles of varying sizes and textures that had absorption peaks around 1100 cm^{-1} appeared on all samples, except for the *1 keV post-25 μL* experiment, the controls, and the standards. There were also particles observed on the surface of all samples that did not peak in the 1100 cm^{-1} range, likely contamination (e.g., round particle on top of lower rod in Fig. S11a). Rod-like particles were observed on the surface of the *5 keV pre-25 μL* sample and showed greater IR amplitudes compared to the surrounding surface (Fig. S11a). Some of these Si-O bond peaked particles also showed an additional peak in the 1175 cm^{-1} range. Rod-like particles were observed in multiple locations for the *5 keV pre-25 μL* sample and in one location on the *5 keV pre-50 μL* sample (~1 to 12 μm in length for rods seen in scans) but were not observed on the remaining samples. Particles with a different shape were observed on the *1 keV pre-25 μL* sample (Fig S11b). These particles appeared to all have the same shape at different size scales, almost like growing crystals, which may be the true particle shape, or the different texture could be due to an AFM tip artifact where the AFM tip shape is convolved with the surface topography.[1] Point spectra also revealed an IR amplitude peak around 1100 cm^{-1}, with one smaller particle also peaking around 1175 cm^{-1}. Particles like these, with greater IR amplitudes around 1100 cm^{-1}, were not observed on the control samples, suggesting that these particles may have formed from the irradiation experiments.

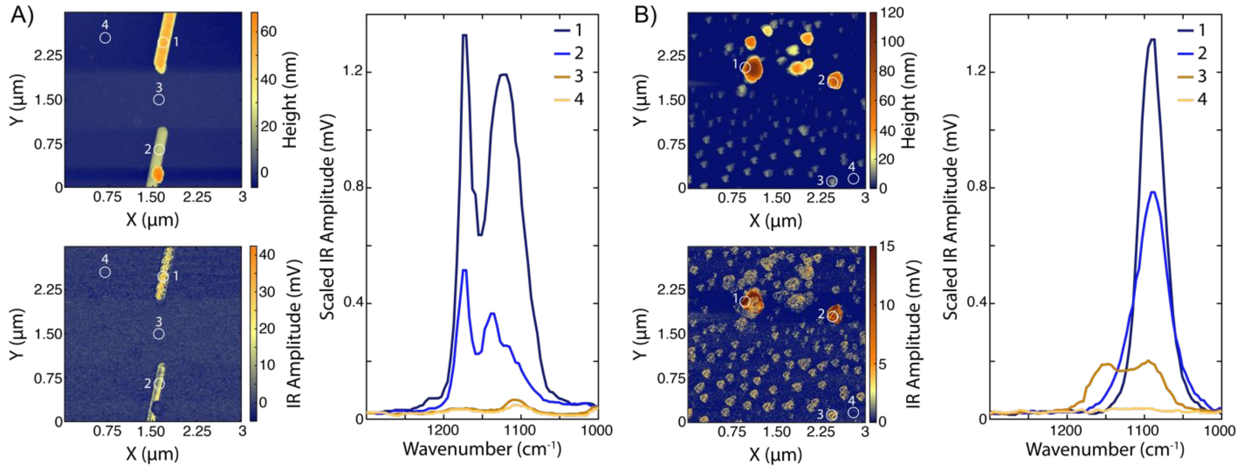

Figure S11. Example 3x3 μm Height and IR Amplitude (1100 cm^{-1}) mapped regions of sample surfaces with particles and corresponding point spectra. A) The *5 keV pre-25 μL* maps show two rod-like particles and the surrounding surface. B) The *1 keV pre-25 μL* maps show particles with similar textures at various sizes.

FIB-S/TEM of a Surface Particle

To further investigate surface particles, a rod from the *5 keV pre-25 uL* sample was FIB sectioned and examined with S/TEM and SEM EDS (Figs. S12-S13). The FIB section process thinned away most of the rod cross section, but a small amount of material remained on the rim, including movement of calcium into the platinum coating. The composition of the rod and comparison spectra suggests the rod is an anhydrite particle (likely a contaminant),[2] which appears similar to Si-O bond spectra within the range of the CSUSM AFM-IR (1931-915 cm^{-1}).

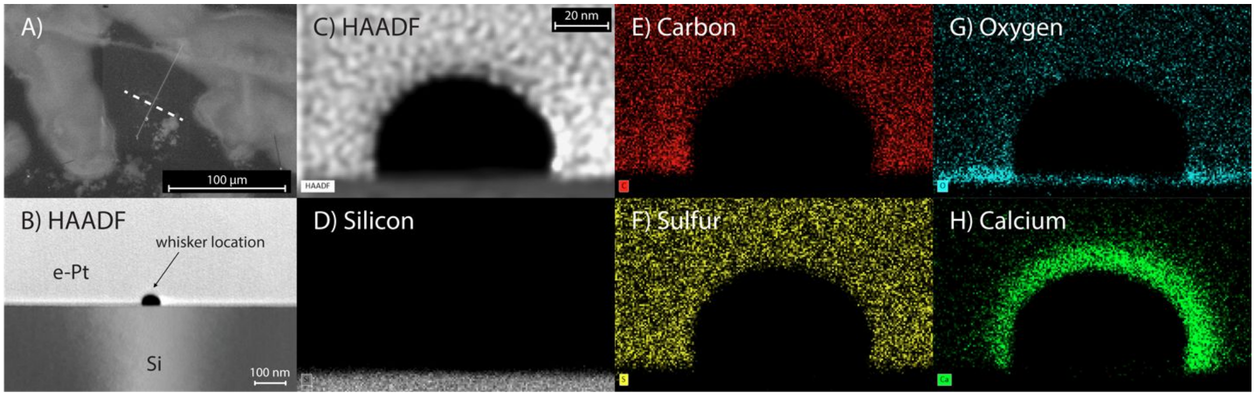

Figure S12. A) SE image of rod with white dashed line showing approximate location of FIB section. B) HAADF image of FIB section with S/TEM. C) Zoom HAADF image, with the possible remnants of the rod around the edge. D-H) EDS maps of rod FIB section. Silicon shows sample surface and lack of silicon in the rod.

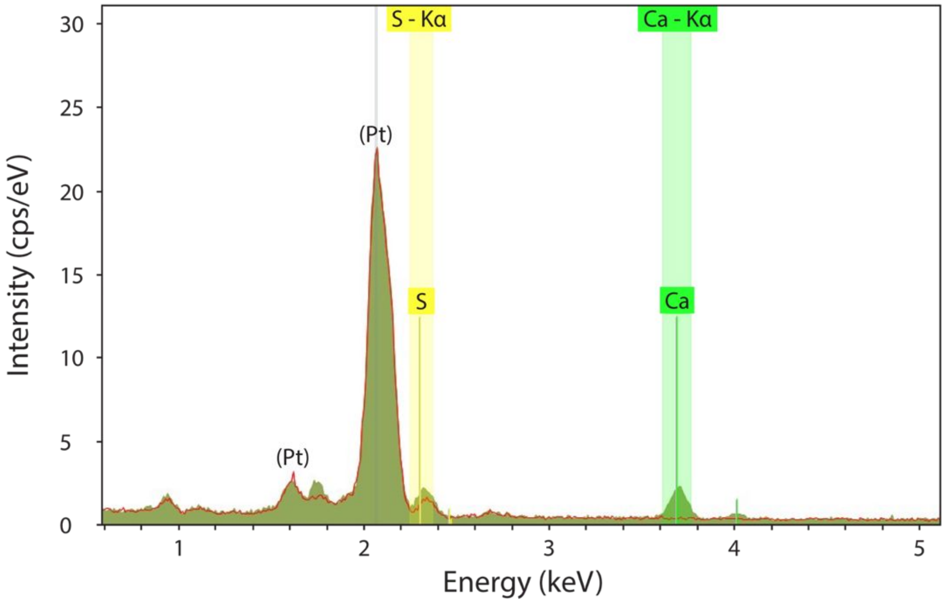

Figure S13. SEM EDS spectra of the remnant rod region with elevated S and Ca (filled green) and a rectangular region of Pt in the upper right of the map (red line) in Fig. S12.

REFERENCES

(1) Gołek, F.; Mazur, P.; Ryszka, Z.; Zuber, S. AFM Image Artifacts. *Applied Surface Science* 2014, *304*, 11–19. https://doi.org/10.1016/j.apsusc.2014.01.149.

(2) Liu, Y.; Wang, A.; Freemen, J. J. Raman, MIR, and NIR Spectroscopic Study of Calcium Sulfates: Gypsum, Bassanite, and Anhydrite. In *40th Annual Lunar and Planetary Science Conference*; 2009; p 2128.
